# The m6A revolution: transforming tumor immunity and enhancing immunotherapy outcomes

**DOI:** 10.1186/s13578-025-01368-z

**Published:** 2025-02-22

**Authors:** Tongguo Shi, Huan Zhang, Yueqiu Chen

**Affiliations:** 1 Jiangsu Institute of Clinical Immunology, The First Affiliated Hospital of Soochow University, 178 East Ganjiang Road, Suzhou, 215000 China; 2 Department of Cardiovascular Surgery of the First Affiliated Hospital and Institute for Cardiovascular Science, Suzhou Medical College of Soochow University, Soochow University, 178 East Ganjiang Road, Suzhou, 215000 China

**Keywords:** Cancer, N6-methyladenosine (m6A), Tumor immunity, Immunotherapy, Immune checkpoint inhibitor

## Abstract

N6-methyladenosine (m6A), the most prevalent RNA modification in eukaryotes, plays a critical role in the development and progression of various diseases, including cancer, through its regulation of RNA degradation, stabilization, splicing, and cap-independent translation. Emerging evidence underscores the significant role of m6A modifications in both pro-tumorigenic and anti-tumorigenic immune responses. In this review, we provide a comprehensive overview of m6A modifications and examine the relationship between m6A regulators and cancer immune responses. Additionally, we summarize recent advances in understanding how m6A modifications influence tumor immune responses by directly modulating immune cells (e.g., dendritic cells, tumor-associated macrophages, and T cells) and indirectly affecting cancer cells via mechanisms such as cytokine and chemokine regulation, modulation of cell surface molecules, and metabolic reprogramming. Furthermore, we explore the potential synergistic effects of targeting m6A regulators in combination with immune checkpoint inhibitor (ICI) therapies. Together, this review consolidates current knowledge on the role of m6A-mediated regulation in tumor immunity, offering insights into how a deeper understanding of these modifications may identify patients who are most likely to benefit from immunotherapies.

## Background

Tumor immunity represents a highly intricate and dynamic component of cancer biology, encompassing both innate and adaptive immune responses [1, 2]. The immune system exerts a pivotal role in recognizing and eliminating emerging tumor cells through the process of cancer immunoediting, which involves both protective and tumor-promoting mechanisms [3]. A deeper understanding of the interactions between different immune cell populations and the tumor microenvironment can furtherance the development of more effective therapeutic strategies, aimed not only at directly targeting tumors but also at reprogramming the immune system to sustain durable antitumor responses [4]. In recent years, cancer immunotherapy approaches, including cancer vaccines, immune checkpoint inhibitors (ICIs), and adoptive cell transfer therapies, have transformed the landscape of cancer treatment [5]. Nevertheless, these immunotherapies demonstrate efficacy in only a subset of cancer patients and are frequently associated with the development of treatment resistance, underscoring the urgent need for novel therapeutic strategies to enhance the efficacy and durability of immune-based cancer treatments [5].

N6-Methyladenosine (m6A), the methylation of adenosine at the N6 position, is the most prevalent RNA modification in mammalian eukaryotic cells [6, 7]. Typically, m6A modifications occur within a conserved consensus sequence, RRACH (where R = G or A and H = A, C, or U), with an enrichment near the 3′ untranslated region (3′ UTR) and stop codons [6, 7]. This modification is a reversible and dynamic process, regulated by three key groups of proteins: “writers” (methyltransferases that catalyze the methylation process), “readers” (binding proteins that recognize and interpret the m6A mark), and “erasers” (demethylases that remove the methyl group) [8, 9]. m6A modification has been recognized as a crucial regulator of RNA metabolism, influencing RNA stabilization, degradation, splicing, and cap-independent translation. Moreover, it has been implicated in the pathogenesis and progression of various diseases, including cancer [6, 10]. Despite its well-established roles, the relationship between m6A modification and tumor immunity, as well as its impact on cancer immunotherapy, has not been comprehensively elucidated.

In this review, we present a current and comprehensive analysis of m6A modifications and their role in modulating immune responses within the tumor microenvironment (TME). Furthermore, we explore how these modifications influence the enhancement of immunotherapy efficacy, offering insights into potential strategies for improving therapeutic outcomes in cancer treatment.

### The process of m6A modification

**Fig. 1:**
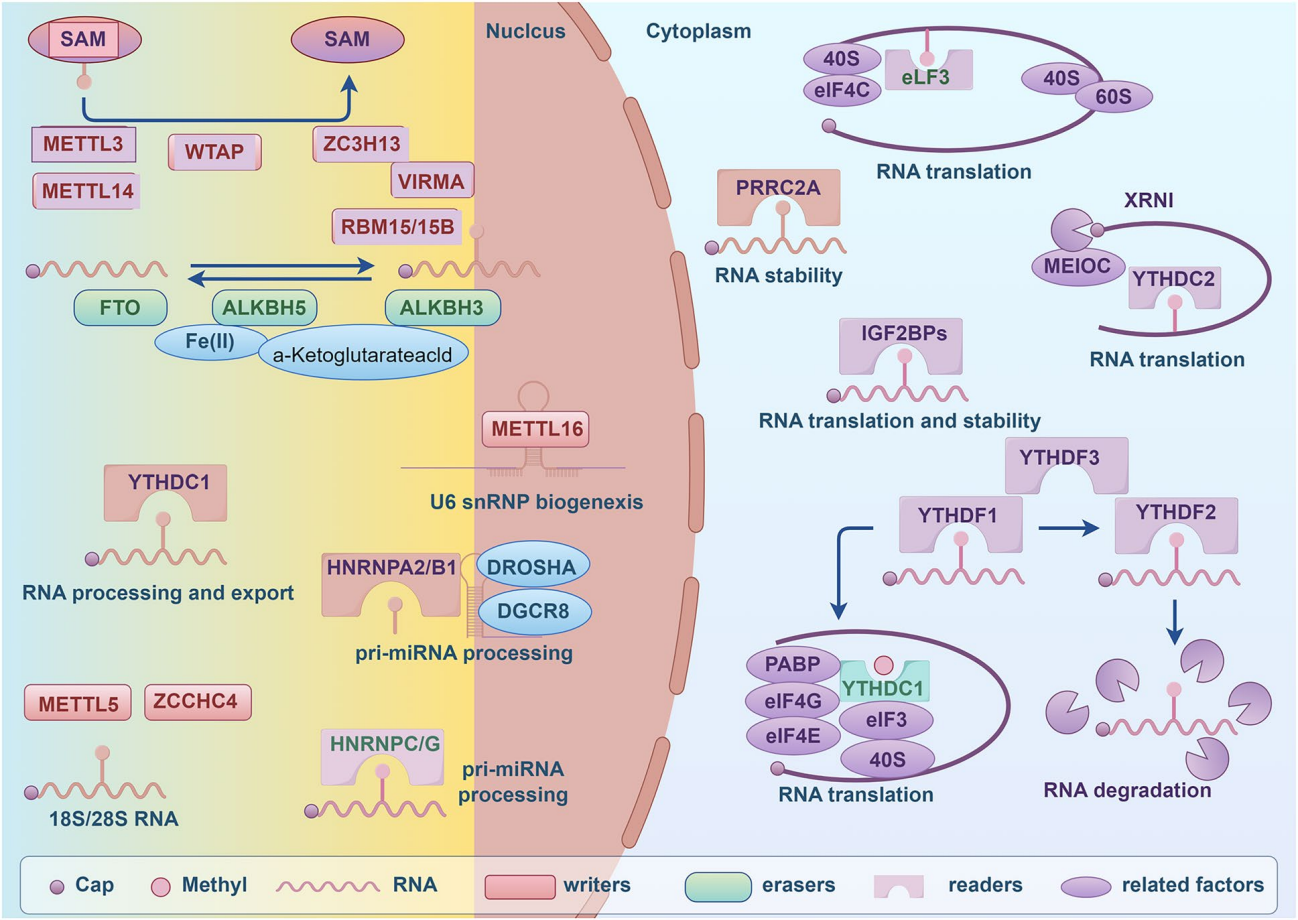
Mechanisms of RNA m6A modification. The graphic was created by Figdraw (www.figdraw.com)

RNA m6A modification is a dynamic and reversible process regulated by several key components, including methyltransferases, demethylases, and methylation-binding proteins (Fig. 1) [11, 12]. The methyltransferase complex, which functions as the “writer” of m6A modifications, consists of methyltransferase-like 3 (METTL3) and methyltransferase-like 14 (METTL14) proteins, along with their associated cofactors: WT1-associated protein (WTAP), RNA-binding motif protein 15/15B (RBM15/15B), Vir-like m6A methyltransferase-associated protein (VIRMA), and zinc finger CCCH-type containing protein 13 (ZC3H13). These components collaboratively catalyze the methylation of adenosine residues, contributing to the regulation of m6A modifications [13]. It is well-established that METTL3, a protein that binds to S-adenosylmethionine (SAM), functions as the catalytic core of the m6A methyltransferase complex, while METTL14 provides structural support and WTAP serves as a stabilizing factor [14, 15]. RBM15/15B facilitates the localization of the complex by binding to METTL3 and WTAP, thereby recruiting the methyltransferase complex to specific RNA target sites [16]. VIRMA directs the complex to the 3′ UTR or stop codon regions of messenger RNA (mRNA) [17]. Additionally, ZC3H13 interacts with WTAP to stabilize the methyltransferase complex within nuclear speckles, enhancing its catalytic activity [18]. Recent studies have identified additional methyltransferases, including METTL16, METTL5, and ZCCHC4 [19–21]. METTL16 plays a critical role in controlling the expression of methionine adenosyltransferase 2A (MAT2A) mRNA and U6 snRNA, thereby maintaining SAM homeostasis and contributing to DNA damage repair [22, 23]. Moreover, METTL5 and ZCCHC4 are involved in the methylation of 18S rRNA and 28S rRNA, respectively, further expanding the scope of RNA modifications in cellular processes [20, 21].

The “erasers” of m6A modifications are enzymes responsible for removing m6A marks from RNA, utilizing Fe2⁺ as a cofactor, with α-ketoglutarate serving as a substrate for the demethylation process [6]. Fat mass and obesity-associated protein (FTO), AlkB homolog 5 (ALKBH5), and ALKBH3 have been identified as key m6A erasers [24]. FTO removes methyl groups from m6A sites, preferentially binding to pre-mRNA in intronic regions, thereby selectively regulating RNA splicing and 3′-end processing [25]. ALKBH5, on the other hand, regulates mRNA export and metabolism by demethylating m6A-modified RNAs [26]. In contrast, ALKBH3 is specifically involved in the removal of m6A from tRNA, rather than mRNA, highlighting its distinct functional role in RNA metabolism [27].

The “readers” of m6A modifications selectively bind to methylated RNAs, affecting RNA fate and mediating specific biological functions [28, 29]. Key m6A reader proteins include YT521-B homology (YTH) domain family proteins (YTHDF1/2/3), YTH domain-containing proteins (YTHDC1/2), heterogeneous nuclear ribonucleoproteins (HNRNPC, HNRNPG, HNRNPA2B1), insulin-like growth factor 2 mRNA-binding proteins (IGF2BP1/2/3), and eukaryotic translation initiation factor 3 (eIF3) [9, 30, 31]. YTHDF proteins, including YTHDF1, YTHDF2, and YTHDF3, function as principal m6A-binding proteins [32, 33]. They regulate m6A-modified mRNA through a unified model, where the effect of YTHDF proteins on m6A-modified mRNAs is proportional to the number of m6A sites [34]. YTHDF1 primarily facilitates mRNA translation, YTHDF2 promotes mRNA degradation, and YTHDF3 performs both functions [32, 33]. Additionally, YTHDC1 and YTHDC2, also YTH domain-containing proteins, play critical roles in RNA processing. YTHDC1 binds to both mRNAs and non-coding RNAs (ncRNAs), aiding in their processing and nuclear export, while YTHDC2 regulates the translation and stability of target genes [35–37]. The heterogeneous nuclear ribonucleoprotein (HNRNP) family, including HNRNPA2B1, HNRNPC, and HNRNPG, represents another group of RNA-binding proteins that function as m6A readers [9, 31]. HNRNPA2B1 enhances primary miRNA processing and regulates alternative splicing and mRNA maturation [38, 39]. Additionally, HNRNPA2B1 acts as a RNA matchmaker to mediate effects of m6A [40]. Although HNRNPC and HNRNPG do not directly bind to m6A sites, they modulate the selective splicing of transcripts by recognizing and interacting with m6A-dependent structural switches [9, 30, 31]. Independent m6A-binding proteins, such as IGF2BP1, IGF2BP2, and IGF2BP3, enhance mRNAs’ translation and stability by specifically recognizing the consensus sequence GG(m6A)C [41, 42]. Eukaryotic translation initiation factor 3 (eIF3) is also regarded as a reader of m6A and plays a vital role in the mRNA translation process [43]. Recently, proline-rich coiled-coil 2A (PRRC2A) has been identified as a novel m6A reader that participates in the regulation of mRNA stabilization [44].

The process of m6A modification is intricate and multifaceted, governed by a variety of regulators categorized as “writers,” “erasers,” and “readers.” Despite this classification, our understanding of the m6A modification process remains limited. For instance, the diversity and functional roles of “writers” warrant further exploration and refinement. Additionally, the functions of m6A “readers” are notably complex and varied, necessitating thorough investigation. The m6A pathway proteins are regulated by multiple mechanisms, including phosphorylation, SUMOylation and caspase-mediated cleavage. For example, Zhang et al. demonstrated that caspase-mediated cleavage of YTHDF2 antagonizes its anti-viral activity during Epstein-Barr virus reactivation process [45]. SUMOylation, a post-translational modification, modulates the stability and function of m6A pathway proteins such as METTL3 and YTHDF2 [46–48]. Exploring these regulatory mechanisms, especially in the context of m6A modification, is crucial.

Overall, the process of m6A modification is an intricate and complex process involving multiple molecules, such as METTL3, FTO and YTHDF1. It has been shown that dysfunctional m6A modification contributes to the development and progression of various malignant tumors [6]. Consequently, a comprehensive understanding of the molecular intricacies associated with m6A modification is essential for elucidating its role in various cancers.

### M6A modification directly regulates immune cell function in TME

Immune cells, including macrophages, natural killer (NK) cells, dendritic cells (DCs), myeloid-derived suppressor cells (MDSCs), and T cells, play a crucial role in tumor progression by functioning within the TME to either suppress or promote cancer development [49–52]. The m6A modification is believed to be involved in various aspects of tumor immunity by modulating these cell populations and their functions, thereby contributing to the establishment of an immunosuppressive TME that enables cancers to evade immune surveillance and destruction [5, 30, 53]. In this context, we highlight recent findings pertaining to these interactions.

### DCs

DCs play a multifaceted role in tumor progression, acting as both promoters and inhibitors of cancer development depending on the specific context [54, 55]. They are essential for initiating immune responses by presenting antigens to T cells; however, their functionality can be compromised within the TME, resulting in immune tolerance and enhanced tumor progression [54, 55]. Recent studies have focused on the regulatory effects of m6A modifications on the activation of DCs within the immune response in the TME.

Wang et al. demonstrated that the targeted depletion of METTL3 in DCs led to impaired phenotypic and functional maturation of these cells, as evidenced by decreased expression of the costimulatory molecules CD40 and CD80, along with reduced production of the cytokine IL-12. Consequently, the capacity of DCs to stimulate T cell responses was significantly diminished in both in vitro and in vivo models [56]. Mechanistically, METTL3-mediated m6A modifications of CD80, CD40, and TIR domain-containing adaptor protein (TIRAP) transcripts in DCs enhanced their translation, thereby upregulating T cell activation and toll-like receptor 4 (TLR4)/NF-κB signaling-induced cytokine production [56]. Conversely, the absence of the m6A reader YTHDF1 in classical DCs increased the capacity of these cells for cross-presentation of tumor antigens and cross-priming of CD8 + T cells by enhancing the translation of mRNAs encoding lysosomal proteases, which facilitate antigen degradation within lysosomes [57]. Collectively, these findings indicate that m6A modifications play significant roles in modulating the activation and cross-presentation of tumor antigens by DCs, thereby offering new therapeutic strategies centered on DCs for cancer treatment.

### Tumor-associated macrophages (TAMs)

**Fig. 2:**
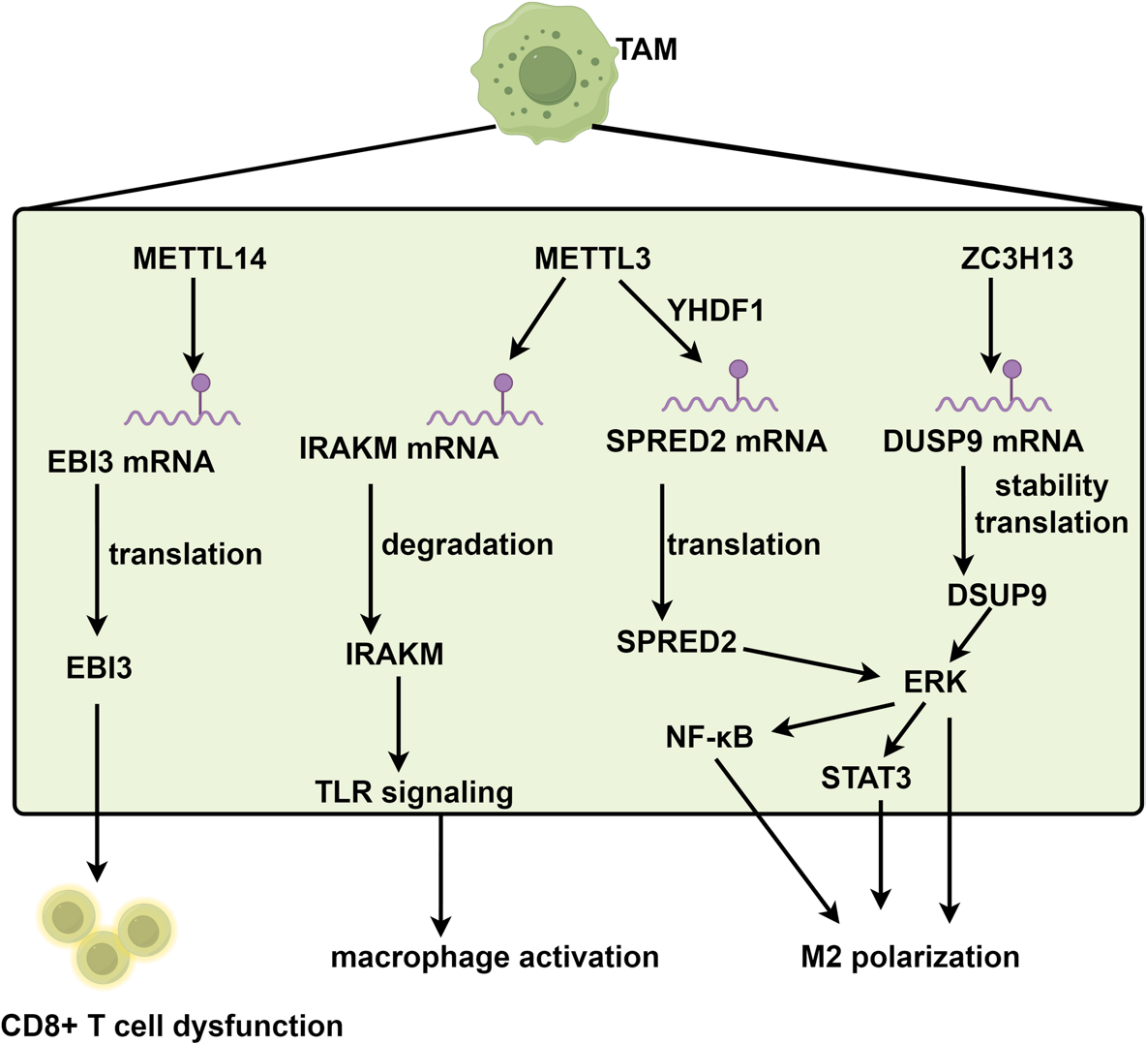
Roles of m6A modifications in modulating macrophage function within the TME. The graphic was created by Figdraw (www.figdraw.com)

TAMs are a critical component of the tumor microenvironment, exhibiting a dual role in both cancer progression and immune response [58, 59]. They display remarkable plasticity, capable of polarizing into either M1 or M2 phenotypes, which exert opposing effects on tumor dynamics [58, 59]. A comprehensive understanding of TAM behavior and manipulation is essential for developing effective cancer therapies [60]. An increasing body of research has established a link between m6A modification and the activation and plasticity of TAMs (Fig. 2). Lihui Dong and colleagues employed single-cell RNA sequencing to identify a C1q + TAM subset in tumors from a Lewis lung carcinoma (LLC) mouse model, characterized by a distinct RNA m6A methylation molecular phenotype [61]. Furthermore, METTL14-deficient TAMs were found to impair the CD8 + T cell infiltration and antitumor response by modulating the m6A methylation of the Epstein-Barr virus-induced 3 (EBI3) transcript [61]. In the context of glioma, neuron-derived exosomal miR-200c-3p was shown to reduce the levels of the m6A writer ZC3H13 in microglia, impairing the methylation of dual specificity phosphatase 9 (DUSP9) mRNA, activating the p-ERK pathway, and ultimately inducing microglial M2 polarization [62]. The deficiency of METTL3 led to the absence of m6A modification on interleukin 1 receptor-associated kinase 3 (IRAK3) mRNA, resulting in its prolonged half-life and elevated levels. This accumulation subsequently inhibited TLR signaling-mediated macrophage activation, thereby facilitating tumor growth in vivo [63]. In addition to its effects on macrophage activation, METTL3 deficiency also influences the reprogramming of these immune cells. Yin et al. observed that mice lacking METTL3 exhibited increased infiltration of M1/M2-like TAMs and regulatory T cells into tumors. Mechanistically, the absence of METTL3 disrupted the YTHDF1-mediated translation of sprouty-related EVH1 domain-containing 2 (SPRED2), enhancing the activation of STAT3 and NF-κB via the ERK pathway. This disruption facilitated the polarization of bone marrow-derived macrophages (BMDMs) into M1 and M2 phenotypes [64]. Overall, these findings underscore the crucial roles of m6A regulators in modulating macrophage function within the TME.

### MDSCs

MDSCs represent a heterogeneous population of immune cells that play a pivotal role in cancer progression by establishing an immunosuppressive tumor microenvironment [65, 66]. These cells are implicated in various malignancies, including lung, ovarian, endometrial, and liver cancers, where they facilitate immune evasion, promote tumor growth, and contribute to treatment resistance [65, 66]. Following exposure to ionizing radiation, the depletion of YTHDF2 in myeloid cells has been shown to enhance antitumor immunity and reduce tumor radioresistance. This effect is mediated by the modulation of MDSC differentiation, as well as the inhibition of their infiltration and suppressive functions [67]. Bone morphogenetic protein and activin membrane-bound inhibitor (BAMBI), a pseudoreceptor for TGF-β, negatively regulates TGF-β signaling and influences cancer progression. YTHDF2 directly binds to and degrades Bambi transcripts in MDSCs in an m6A-dependent manner, thereby affecting both the tumor-infiltrating capacity and suppressive function of MDSCs through the inhibition of TGF-β signaling [68]. Additionally, lactate-derived lactylation of histone lysine residues represents an epigenetic modification that directly promotes gene transcription from chromatin [69]. Xiong et al. demonstrated that H3K18 lactylation upregulates METTL3 in tumor-infiltrating myeloid cells (TIMs). Moreover, METTL3 enhances m6A methylation on Janus kinase 1 (JAK1) mRNA in TIMs, promoting JAK1 protein translation and subsequent phosphorylation of STAT3, which further amplifies the immunosuppressive capacity of TIMs [70]. In summary, m6A methylation is likely to mediate the infiltration and functional dynamics of MDSCs in tumors following radiotherapy. However, further investigation is warranted to elucidate how m6A methylation influences tumor progression and therapy through its effects on MDSCs.

### NK cells

NK cells are a crucial component of the innate immune system, with a well-established role in both antiviral and antitumor responses [71, 72]. These cells utilize a range of mechanisms to identify and eliminate virus-infected and malignant cells [71, 72]. Recent attention has been drawn to the significance of m6A RNA modification in regulating NK cell-mediated antitumor immunity. Ma et al. demonstrated that YTHDF2 expression is elevated in NK cells upon activation by cytokines, tumors, and cytomegalovirus infection. Furthermore, the ablation of YTHDF2 in NK cells impairs their antitumor and antiviral functions by destabilizing the mRNA of TAR DNA-binding protein (TARDBP) and modulating the activity of signal transducer and activator of transcription 5 (STAT5) and Eomesodermin (Eomes) [73]. In a separate study, Song et al. noted a positive correlation between METTL3 expression and effector molecule production in tumor-infiltrating NK cells. Deletion of METTL3 in NK cells led to reduced infiltration and impaired function in the TME, due to diminished SHP-2 levels, which in turn suppressed activation of the MAPK and AKT signaling axis in an m6A-dependent manner [74]. However, the roles of other m6A regulators in NK cell function and cancer progression remain largely unexplored.

### T cells

T cells are the primary effector cells in cellular immunity, playing a critical role in combating viral infections and eliminating tumor cells [75]. T cell-based cancer immunotherapy, which exploits their capacity to recognize and destroy cancer cells, has demonstrated considerable therapeutic potential [76, 77]. Enhancing T cell function within the TME has emerged as a promising strategy to inhibit tumor progression [76, 77]. Increasing evidence highlights the role of m6A RNA modification in regulating T cell homeostasis and function. For example, the deletion of METTL3 in CD4 + T cells disrupts their homeostasis and differentiation by impairing IL-7-mediated STAT5/suppressor of cytokine signaling (SOCS) activation [78]. Additionally, it compromises the function and stability of Treg cells by inhibiting IL-2/STAT5 signaling, while simultaneously enhancing T effector cell cytokine secretion and promoting antitumor immune responses within the TME [79]. Furthermore, deubiquitinase ubiquitin-specific peptidase 47 (USP47) was found to prevent YTHDF1 ubiquitination, thereby disrupting its interaction with the translation initiation machinery and reducing m6A-mediated c-MYC translation efficiency, which is crucial for maintaining Treg cell metabolic and functional homeostasis [80]. Among T cell subtypes, γδ T cells also exert an essential role in cancer immunity [81]. In our recent research, we found that exosomal Thrombospondin 1 (THBS1) derived from gastric cancer (GC) cells regulates METTL3- or IGF2BP2-mediated m6A modification, activating the RIG-I-like receptor signaling pathway in Vγ9Vδ2 T cells, thereby enhancing their cytotoxicity against GC cells [82].

### B cells

B cells (B lymphocytes), an important type of white blood cell in the immune system, possess a specific receptor known as the B cell receptor (BCR) and are primarily responsible for humoral immune responses [83, 84]. B cells can directly recognize antigens, as well as take up, process, and present them to T cells, particularly CD4 + helper T cells [83, 84]. Recent studies have highlighted the crucial and synergistic role of B cells in tumor control [85] and the involvement of m⁶A modifications in regulating B cells in cancers. For instance, YTHDC1 is highly expressed in B-cell acute lymphoblastic leukemia (B-ALL), where it binds to and stabilizes m⁶A-modified KMT2C mRNA. This interaction increases histone H3K4 methylation and the expression of DNA damage response (DDR)-related genes, ultimately leading to a decreased DNA damage response in B-ALL [86]. Additionally, Meng et al. demonstrated that nuclear cap-binding protein 1 (NCBP1) enhances the m⁶A catalytic function of METTL3, increasing c-MYC expression and promoting the proliferation of diffuse large B-cell lymphoma (DLBCL) [87]. Furthermore, studies have shown that Protein arginine methyltransferase 5 (PRMT5)-deficient mouse B cells exhibit dysregulated distribution of RNA m⁶A modifications, which slows colorectal tumor progression [88]. These findings indicate that m⁶A modification exerts complex regulatory roles in B cell-mediated tumor immune responses. Therefore, developing targeted therapeutic drugs against key proteins such as YTHDC1 and METTL3, based on the regulatory mechanisms of m⁶A modification, holds promise for enhancing the anti-tumor functions of B cells.

Collectively, m6A modification, one of the most significant epigenetic regulators, plays a crucial role in the function of various immune cells within the TME. It is particularly important to underscore the influence of m6A modification on the interactions between cancer cells and immune cells. In the following section, we will focus on the effects of m6A modification on mediating these interactions.

### Role of m6A methylation in the regulation of interactions between cancer cells and immune cells

Recent studies have shown that aberrant m6A RNA modification in cancer cells significantly impacts the infiltration and function of various immune cells, including T cells, TAMs, and MDSCs, thereby resulting in the development of an immunosuppressive TME [89]. In this context, we provide an overview of the role of m6A modification in modulating the interactions between cancer cells and immune cells.

### m6A modification regulates immune respones via cytokines

**Fig. 3:**
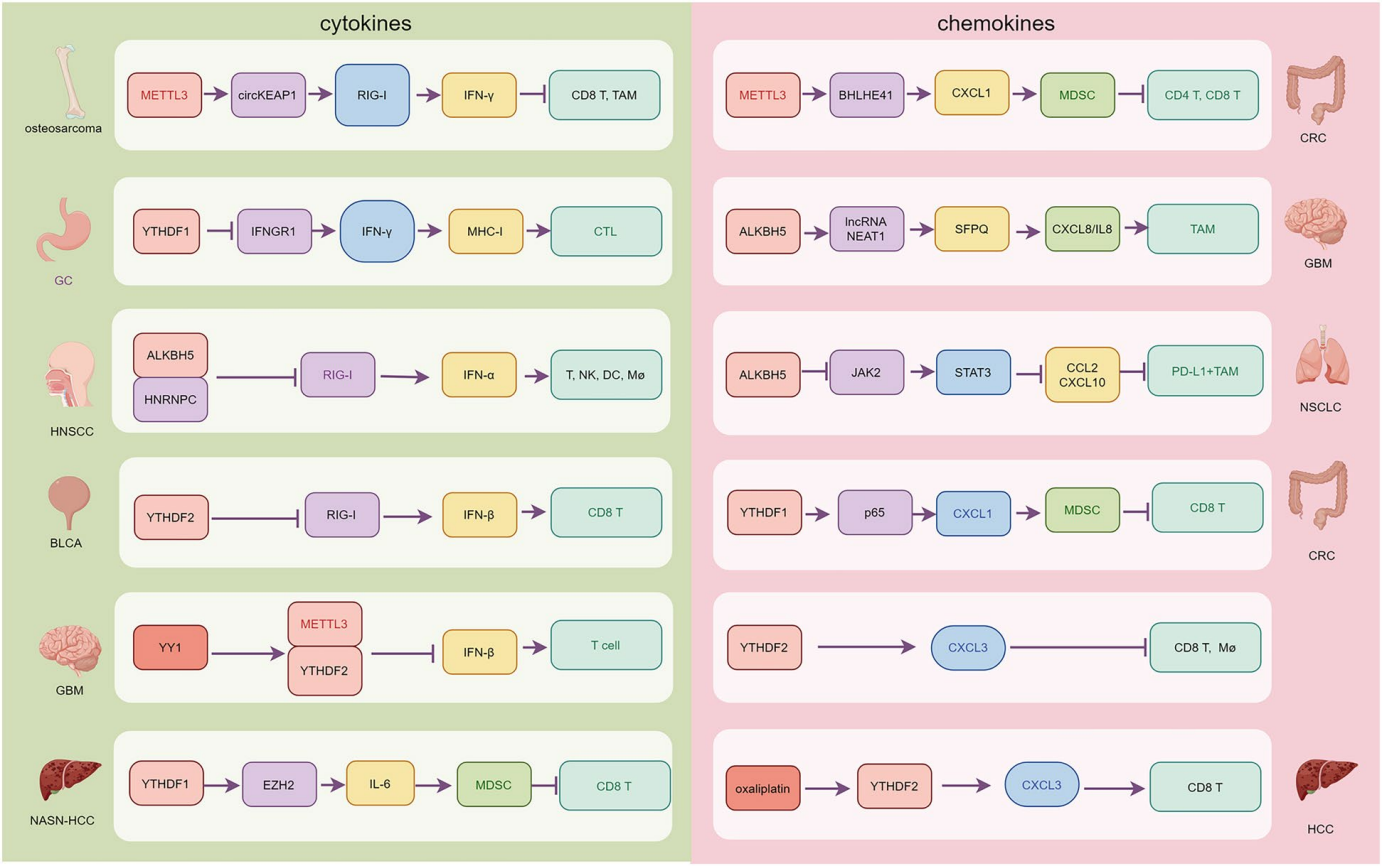
m6A modification regulates the interactions between cancer cells and immune cells through the cytokones and chemokines. The graphic was created by Figdraw (www.figdraw.com)

It is well established that type II interferon (e.g., IFN-γ) and type I interferons (e.g., IFN-α and IFN-β) are key mediators of the interactions between cancer cells and immune cells within the TME [90, 91]. Increasing evidence suggests that m6A RNA modification plays a critical role in regulating the production of IFNs in cancer cells, thereby influencing the immune response in the TME (Fig. 3) [5]. IFN-γ, a pleiotropic cytokine, is particularly important in cellular immunity and the stimulation of antitumor immune responses [92]. Loss of YTHDF1 has been shown to mediate overexpression of IFN-γ receptor 1 (IFNGR1) in GC cells, enhancing the IFN-γ response and promoting the expression of major histocompatibility complex class I (MHC-I) on tumor cells, facilitating the presentation of immunogenic tumor cells to cytotoxic T lymphocytes (CTLs) and triggering strong antitumor responses [93]. In another study, Zhang et al. identified m6A in circKEAP1, demonstrating that decreased m6A modification induced by METTL3 reduced circKEAP1 expression and stability in osteosarcoma cells. Overexpression of circKEAP1 interacted with RIG-I, thereby enhancing antitumor immunity via the IFN-γ pathway [94]. Type I IFNs, including IFN-α and IFN-β, exert direct effects on cancer cells and indirect effects through immune effector cells and the vasculature [95]. Jin et al. uncovered a novel ALKBH5/RIG-I/IFN-α axis, demonstrating that m6A-dependent binding of HNRNPC to DDX58 mRNA (which encodes RIG-I) promotes tumor immune evasion in head and neck squamous cell carcinoma (HNSCC) by facilitating immune escape [96]. Furthermore, loss of YTHDF2 in bladder cancer (BLCA) cells activated an innate immune response, enhancing CD8 + T cell infiltration into the TME. Mechanistically, YTHDF2 binds to the coding region of DDX58 mRNA and mediates its degradation in an m6A-dependent manner, leading to upregulation of IFN-β expression [97]. In glioblastoma (GBM) stem cells, METTL3 and YTHDF2 have been implicated in Yin Yang 1 (YY1)-mediated IFN-β signaling and antigen presentation through m6A methylation, reducing Treg cell infiltration and improving the efficacy of immune checkpoint therapy [98]. In a word, m6A modifications promote antitumor immune responses through the regulation of IFNs. Enhancing m6A-mediated IFN signaling may represent a novel approach to improving cancer immunotherapy.

In addition to IFNs, other cytokines, such as IL-6, are also involved in m6A modification-mediated tumor immune responses. Utilizing single-cell RNA sequencing, Wang et al. and colleagues demonstrated that YTHDF1 promotes the accumulation of MDSCs and suppresses cytotoxic CD8 + T cell function in the tumor tissues of spontaneous non-alcoholic steatohepatitis-associated hepatocellular carcinoma (NASH-HCC) models [99]. Mechanistically, YTHDF1 binds to m6A-modified enhancer of zeste homolog 2 (EZH2) mRNA, facilitating its translation. This upregulates IL-6 expression and secretion, which in turn recruits and activates MDSCs, leading to CD8 + T cell dysfunction [99]. However, further research is needed to elucidate the involvement of additional cytokines in m6A modification-mediated immune responses within the TME.

### m6A modification regulates immune respones via chemokines

Chemokines are chemotactic cytokines that regulate immune cell migration and function as a double-edged sword in tumor immune responses [100]. m6A RNA modifications have been implicated in the regulation of chemokines within cancer cells, influencing the infiltration of immune cells into the TME (Fig. 3). In colorectal cancer (CRC), knockdown of METTL3 disrupted the m6A-dependent basic helix-loop-helix family member e41 (BHLHE41)-CXCL1 axis, leading to reduced accumulation of MDSCs, while promoting sustained activation and proliferation of CD4 + and CD8 + T cells, ultimately suppressing CRC progression [101]. Similarly, the m6A demethylase ALKBH5 plays a role in modulating immune responses in TME via chemokine signaling. Silencing or inactivating ALKBH5 in GBM cells significantly reduced hypoxia-induced recruitment of TAMs and immunosuppression by regulating the m6A- lncRNA NEAT1-splicing factor proline and glutamine rich (SFPQ)-CXCL8/IL8 signaling pathway [102]. In non-small-cell lung cancer (NSCLC), ALKBH5 mediated m6A modification of *JAK2* mRNA, activated the JAK2/p-STAT3/CCL2/CXCL10 axis, leading to the recruitment of PD-L1 + TAMs and promoting M2 macrophage polarization [103]. Recent studies have also reported the involvement of m6A readers YTHDF1 and YTHDF2 in chemokine-induced immune responses within the TME. Overexpression of YTHDF1 enhanced the translation of p65 in CRC cells in an m6A-dependent manner, with the YTHDF1/p65 axis upregulating MDSC migration via the CXCL1-CXCR2 axis, thereby antagonizing functional CD8 + T cells [104]. Conversely, loss of YTHDF2 in tumor cells led to macrophage recruitment via CX3CL1 and enhanced mitochondrial respiration in CD8 + T cells by impairing tumor glycolysis [105]. Interestingly, in peritumoral hepatocytes, YTHDF2 facilitated oxaliplatin-induced antitumor immune responses by stabilizing C CX3CL1 transcripts in an m6A-dependent manner, promoting CD8 + T cell recruitment and activation [106].

### m6A modification regulates immune respones via cell surface molecules

Tumor cells express surface molecules critical to tumor immunity, including immune checkpoints such as PD-L1, CTLA-4, and V-domain Ig suppressor of T cell activation (VISTA) [107, 108]. These molecules play a key role in regulating antitumor immunity by either co-stimulating or co-inhibiting the cytotoxic functions of T cells, making them important targets for immunotherapeutic strategies [107, 108]. Emerging evidence indicates that m6A RNA modification modulates immune responses in the TME through its regulation of these checkpoint molecules.

PD-L1, also known as CD274, is a prominent inhibitory checkpoint molecule that controls T cell activity by interacting with its receptor, PD-1 [109]. Tumor cells exploit various molecular mechanisms, including m6A modification, to upregulate PD-L1 expression and evade T cell-mediated immunity [110, 111]. Moreover, silencing of METTL3 significantly reduced m6A modification in breast cancer (BC) cells, decreased PD-L1 mRNA stability, and inhibited PD-L1 expression via the m6A-IGF2BP3 axis, enhancing antitumor immunity by promoting T cell activation, infiltration, and reducing exhaustion [112]. In BLCA cells, downregulation of METTL3 reduced m6A modification in PD-L1 mRNA, thereby decreasing its stabilization by IGF2BP1, which enhanced the cytotoxicity of CD8 + T cells against tumor cells [113]. Similarly, knockdown of ALKBH5 in intrahepatic cholangiocarcinoma (ICC) cells increased m6A modifications in the 3′UTR of PD-L1 mRNA, resulting in its degradation via YTHDF2. This resulted in reduced MDSC infiltration and enhanced antitumor T cell immunity [114]. Moreover, both methionine-restricted diets and YTHDF1 knockdown were shown to reduce m6A methylation and translation of immune checkpoints, including PD-L1 and VISTA, in cancer cells, thereby restoring CD8 + T cell infiltration into the tumor [34]. Collectively, these findings suggest that m6A-associated proteins directly regulate PD-L1 expression in cancer cells in an m6A modification-dependent manner, highlighting their potential as targets in immunotherapy.

**Fig. 4:**
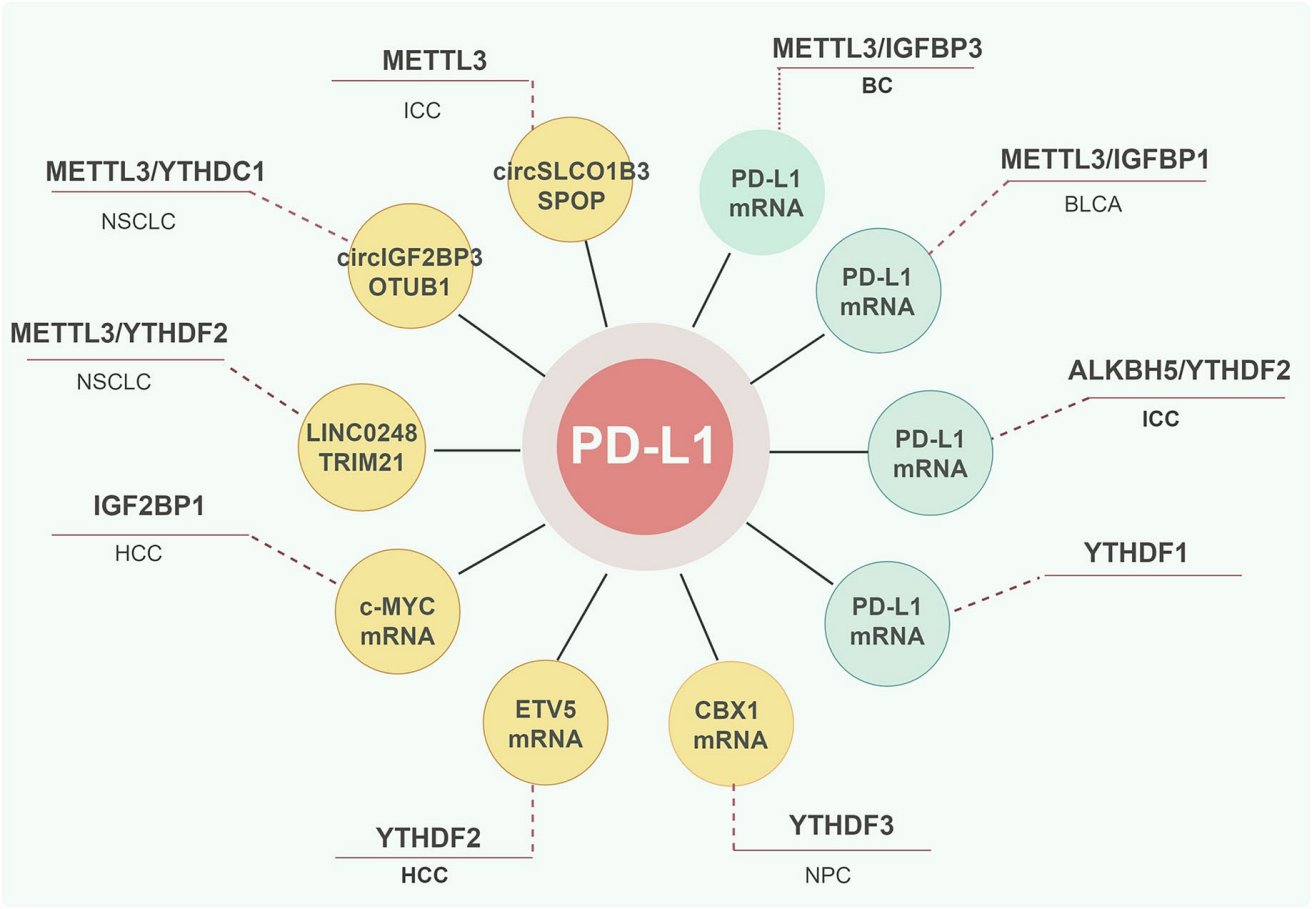
m6A modification modulates PD-L1 expression in cancer cells. The graphic was created by Figdraw (www.figdraw.com)

m6A modification can indirectly regulate PD-L1 expression in tumor cells by influencing various genes and signaling pathways. Chromobox 1 (CBX1), a histone methylation regulator, has been identified as significantly upregulated with m6A hypomethylation in metastatic nasopharyngeal carcinoma (NPC) tissues [115]. The m6A-modified CBX1 mRNA is recognized and destabilized by the m6A reader YTHDF3. CBX1 downregulates the proportion of tumor-infiltrating CD8 + T cells and TNFα + CD8 + T cells through the IFN-γ-STAT1 signaling pathway, leading to the upregulation of PD-L1 [115]. In hepatocellular carcinoma (HCC), YTHDF2 facilitates the m6A-dependent translation of ETS variant transcription factor 5 (ETV5), which induces PD-L1 transcription and suppresses CD8 + T-cell-mediated antitumor immunity [116]. Furthermore, Yang Liu and colleagues reported that IGF2BP1-mediated stabilization of c-MYC mRNA reduced PD-L1 expression in HCC cells, significantly enhancing immune cell infiltration, including CD4 + and CD8 + T cells, CD56 + NK cells, and F4/80 + macrophages [117]. In NSCLC, LINC02418, a negative regulator of PD-L1 expression, was positively correlated with infiltration of CD8 + T cells. Inhibition of METTL3 via m6A modification, mediated by YTHDF2, upregulated LINC02418, leading to reduced PD-L1 expression and enhanced T cell-mediated apoptosis via E3 ligase tripartite motif containing 21 (TRIM21) [118]. Additionally, circIGF2BP3 was found to contribute to immune evasion in NSCLC cells by reducing PD-L1 ubiquitination and preventing its proteasomal degradation. This occurred through the stabilization of ubiquitin aldehyde binding 1 (OTUB1) mRNA in a plakophilin 3 (PKP3)-dependent manner, with METTL3 mediating the m6A modification and circularization of circIGF2BP3 in a YTHDC1-dependent manner [119]. In ICC, METTL3-induced m6A methylation of circSLCO1B3 stabilized its expression, which impaired antitumor immunity by suppressing the ubiquitin–proteasome-dependent degradation of PD-L1 by the E3 ubiquitin ligase speckle type BTB/POZ protein (SPOP) [120]. These studies collectively suggest that m6A modification controls PD-L1 expression and tumor immunity through a variety of mechanisms involving oncogenes, lncRNAs, and circRNAs (Fig. 4).

Beyond PD-L1, m6A modification also influences the tumor immune response through other cell surface molecules. One well-established mechanism of immune evasion is the downregulation or loss of antigens or MHC-I molecules, which are responsible for presenting antigens to T cells [121]. Lin et al. demonstrated that the depletion of YTHDF1 regulated the translation of lysosomal genes, limiting lysosomal proteolysis of MHC-I molecules and antigens. This mechanism ultimately restored the infiltration and cytotoxicity of CD8 + T cells within the TME [122]. CD58, a ligand of CD2 expressed on T lymphocytes, enhances T cell activation [123]. Overexpression of heat shock protein family A (Hsp70) member 4 (HSPA4) in GC cells increased the levels of ALKBH5, which negatively regulated CD58 expression via m6A RNA demethylation, thereby inhibiting the cytotoxic activity of CD8 + T cells [124]. Similarly, leukocyte immunoglobulin-like receptor B4 (LILRB4), a member of the leukocyte Ig-like receptor superfamily, is associated with monocytic leukemia and facilitates tumor cell infiltration into tissues while suppressing T cell activity [125, 126]. Su et al. found that both pharmacological inhibition and genetic depletion of FTO enhanced the sensitivity of acute myeloid leukemia cells to T cell-mediated cytotoxicity and counteracted immune evasion induced by hypomethylating agents, particularly through the downregulation of immune checkpoint genes such as LILRB4 [127]. These findings underscore the broad regulatory role of m6A modification in modulating surface molecules involved in immune responses, beyond PD-L1, thus influencing tumor-immune interactions.

### m6A modification regulates immune respones via energy metabolism reprogramming

Metabolic reprogramming is a hallmark of malignant tumors, playing a pivotal role in sustaining tumorigenesis and progression by altering cancer signaling pathways [128, 129]. Crucially, this reprogramming has been shown to influence the tumor immune responses through the release of various metabolites, including lactate, prostaglandin E2 (PGE2), and arginine, which affect the immune landscape within the TME [129]. Recent studies have increasingly focused on the role of m6A modification in mediating the interconnected metabolic reprogramming of both tumor and immune cells within the TME, highlighting its significance in cancer progression and immune modulation.

Aerobic glycolysis, commonly referred to as the Warburg effect, provides cancer cells with a growth advantage by supplying energy and biosynthetic precursors necessary for proliferation [130]. Additionally, aerobic glycolysis plays a critical role in establishing immunosuppressive networks within the TME [131]. Emerging research has demonstrated that m6A modification regulates aerobic glycolysis in cancer cells, thereby influencing immune cell function in the TME. For instance, FTO-mediated m6A demethylation in tumor cells enhances the expression of transcription factors c-Jun, JunB, and C/EBPβ, which rewires glycolytic metabolism. Silencing of FTO reduces glycolytic activity in tumor cells, leading to the restoration of CD8 + T cell function and inhibition of tumor growth [132]. Phosphoglycerate mutase 1 (PGAM1), an enzyme involved in glycolysis, converts 3-phosphoglycerate to 2-phosphoglycerate [133]. In CRC, METTL3-mediated m6A modification on circQSOX stabilizes it via IGF2BP2, increasing circQSOX expression in CRC cells. This elevated circQSOX level promotes intratumoral infiltration of Treg cells while reducing CD8 + T cell infiltration by sponging miR-330–5p and miR-326 to increase PGAM1 expression and lactate production [134]. Hexokinases (HKs), which modulate the first step of glycolysis—phosphorylation of glucose to glucose-6-phosphate—are critical enzymes in this pathway [135]. Among HK isoenzymes, HK3 is highly expressed in renal cell carcinoma (RCC) tissues [136]. Li et al. reported that HNRNPC facilitated the interaction between circZBTB44 and IGF2BP3 via m6A modification, and circZBTB44 recruited IGF2BP3 to enhance the mRNA stability of HK3. Moreover, circZBTB44 promoted M2 macrophage polarization in RCC by upregulating HK3 [137]. These findings suggest that m6A modification is a key regulator of enzymes involved in aerobic glycolysis in cancer cells, which in turn modulates immune cells such as CD8 + T cells, Treg cells, and M2 macrophages.

Cholesterol metabolism is frequently dysregulated in various cancers, and cholesterol-associated metabolites have been identified as key regulators of tumor immunity [138]. In non-alcoholic fatty liver disease-hepatocellular carcinoma (NAFLD-HCC), METTL3 enhances m6A methylation on the mRNA of sterol regulatory element-binding protein (SREBP) cleavage-activating protein (SCAP), promoting its translation and activating cholesterol biosynthesis. This metabolic alteration impairs the CD8 + T cell function in the TME through elevated levels of cholesterol and cholesteryl esters [139]. These insights into metabolic reprogramming underscore the significance of m6A modification in regulating energy metabolism within cancer. Nevertheless, further research is necessary to investigate the influence of m6A modification on other metabolic pathways, such as fatty acid and amino acid metabolism, and their impact on tumor immune responses.

### m6A modification regulates immune respones via other mechanisms

In addition to cytokines, chemokines, cell surface molecules, and energy metabolism reprogramming, other mechanisms contribute to the impact of m6A modification on tumor immunity. For instance, as a secretory antagonist of the classical Wnt signaling pathway, Dickkopf-1 (DKK1) has been linked to tumor immunity [140]. In CRC models, ALKBH5 promotes the accumulation of MDSCs while reducing NK cells and cytotoxic CD8 + T cells, facilitating tumorigenesis via the Wnt/β-catenin/DKK1 axis by downregulating Wnt suppressor AXIN2 through m6A RNA demethylation [141]. Furthermore, Chen et al. demonstrated that FTO depletion in HCC cells elevated the activation and recruitment of tumor-infiltrating CD8 + T cells by inhibiting the interaction between glycoprotein non-metastatic melanoma protein B (GPNMB) and the CD8 + T cell surface receptor syndecan 4 (SDC4). Mechanistically, FTO increased m6A levels on GPNMB mRNA, stabilizing it from degradation by the YTHDF2 [142]. These data highlight the important crosstalk between m6A modifications and tumor immune responses, underscoring its potential clinical significance.

### m6A regulators act as biomarkers for cancer immune response

The involvement of m6A regulators in immune responses to tumors renders them particularly significant as potential biomarkers for cancer-related immune responses [143, 144]. Chong et al. established a scoring system known as the m6Sig score, which quantifies the m6A modification patterns in individual cases of colon cancer based on identified m6A-related signature genes [145]. Patients with colon cancer exhibiting a lower m6Sig score demonstrated prolonged survival, enhanced immune infiltration, and a correlation with increased tumor mutation burden, PD-L1 expression, and higher mutation rates in significant mutation genes (SMGs) such as PIK3CA and SMAD4 [145]. Similarly, investigations into m6A modification patterns have revealed a significant association between m6Ascore and the tumor immune landscape in patients with GC and clear cell renal cell carcinoma (ccRCC) [146, 147]. Consequently, we further explore the potential of m6A regulators as biomarkers for cancer immune response (Table 1).

**Table 1:** The correlation between m6A regulators and immune indexex in cancer tissues

Name	Cancer type	Marker type	Sample size	Prognostic value	Association	Refs.
METTL3	CRC	Protein	167		Positive correlation with CD33 + MDSC infiltration	[101]
METTL3/IGF2BP3	BC	Protein	140		Positive correlation with PD-L1 expression	[112]
METTL14	CRC	mRNA	22		Positive correlation with CD8 + T cell infiltration	[61]
ALKBH5	GBM	Protein	39		Positive correlation with CD68 + TAM infiltration	[102]
ALKBH5	NSCLC	Protein	55		Positive correlation with PD-L1 and TAM infiltration	[103]
ALKBH5	HNSCC	Protein	138		Negative correlation with the RIG-I and IFN-α	[96]
ALKBH5	ICC	Protein	127		Positive correlation with PD-L1 expression	[114]
ALKBH5	CRC	Protein	775	Poor prognosis		[141]
FTO	HCC	mRNA/Protein	95	Poor overall and disease-free survival		[142]
YTHDF1	GC	Protein/mRNA	278/101	Poor survival		[148]
YTHDF1	CRC	Protein	408	Poor survival	Negative correlation with the IFN-γ response pathway and CD8 + T cell infiltration	[104]
YTHDF1	CRC	Protein	200		Negative correlation with CD8 + T cell infiltration	[149]
YTHDF1	Melanoma	Protein	48		Negative correlation with CD8 + T cell infiltration	[122]
YTHDF2	BLCA	Protein	128	Poor overall survival		[97]
YTHDF2	BLCA	Protein	68		Negative correlation with the RIG-I	[97]
IGF2BP1	HCC	Protein	90			[117]

*CRC* colorectal cancer; *MDSC* Myeloid-derived suppressor cell; *BC* breast cancer; *GBM* glioblastoma; *NSCLC* non-small-cell lung cancer; *HNSCC* head and neck squamous cell carcinoma; *TAM* tumor-associated macrophage; *ICC* intrahepatic cholangiocarcinoma; *HCC* hepatocellular carcinoma; *GC* gastric cancer; *BLCA* bladder cancer; *RIG-I* RNA sensor RIG-I

In patients with CRC, the expression of the METTL3 protein exhibited a significant correlation with the infiltration of CD33 + MDSCs in tumor tissues [101]. Furthermore, higher levels of METTL3 and IGF2BP3 expression were observed in PD-L1-positive BC tissues [112]. Additionally, another m6A methyltransferase, METTL14, along with m6A levels in tumor stromal cells, was found to be associated with dysfunctional T cell levels in CRC patients [61]. A growing body of evidence suggests that demethylases, including ALKBH5 and FTO, are linked to the survival of cancer patients and the infiltration of immune cells within tumor tissues. For instance, Zhai et al. explored the clinical significance of ALKBH5 in CRC patients and identified high ALKBH5 protein expression as an independent poor prognostic factor for CRC. Moreover, elevated ALKBH5 expression correlated with reduced infiltration of CD8 + T cells in CRC tissues [141]. An inverse correlation was also noted between ALKBH5 levels and the expression of the RNA sensor RIG-I and IFNα protein in tissue specimens from patients with HNSCC [96], indicating a negative correlation between ALKBH5 level and antitumor immunity. In clinical samples of GBM, a significant positive correlation between ALKBH5 and CD68 + TAMs was observed [102]. Furthermore, ALKBH5 level positively correlated with PD-L1 expression and macrophage infiltration in NSCLC patients [103]. In a cohort of ICC patients, tumors with high levels of ALKBH5 also demonstrated strong PD-L1 expression [114], suggesting that ALKBH5 plays a role in creating an immunosuppressive microenvironment. Additionally, FTO, another m6A demethylase, is upregulated in HCC tumors. Importantly, HCC patients exhibiting high FTO expression had worse overall and disease-free survival compared to those with low FTO expression [142].

In addition to the writers and erasers of m6A modification, the readers, including YTHDF1, YTHDF2, and IGF2BP1, may represent potential biomarkers for responses to cancer immunotherapies. Bai et al. reported that elevated levels of YTHDF1 in GC tissues are associated with a poor prognosis for GC patients [148]. Similarly, YTHDF1 has demonstrated prognostic significance in the cancer tissues of patients with CRC [104]. Notably, the protein levels of YTHDF1 exhibit a negative correlation with the infiltration of CD8 + T cells in tumor tissues of CRC and melanoma patients [104, 122, 149]. Furthermore, YTHDF2 is significantly upregulated in BLCA tissues, with lower expression levels of YTHDF2 correlating with improved outcomes for BLCA patients [97]. Additionally, Liu et al. found that IGF2BP1 expression is elevated in liver tissues of patients with HCC compared to adjacent normal tissues [117]. Accordingly, these m6A regulators may function as prognostic biomarkers and could reflect the antitumor immune response.

### Targeting m6A regulators improves the response to ICI immunotherapy

Cancer immunotherapies, including checkpoint inhibitors and adoptive cell therapy, have emerged as a formidable clinical strategy for the treatment of cancer [150, 151]. The clinical application of ICIs, such as anti-CTLA-4 and anti-PD-1/PD-L1 antibodies, across various cancer types represents a significant advancement in oncological therapeutics [152]. However, accumulating evidence suggests that ICIs are not universally effective; rather, they demonstrate efficacy in a limited subset of cancer patients who exhibit defects in cancer antigen-specific T-cell activation or impaired T-cell infiltration into tumors [153]. Given that m6A modifications exert critical effects on immune cell infiltration and function within the TME, targeting m6A regulators may represent an effective strategy to enhance the efficacy of immune checkpoint inhibitor therapy (Table 2).

**Table 2:** Targeting m6A regulator improves the response to ICI immunotherapy

M6A regulator	Malignancy	ICB	drugs	Tumor model	Effects/observations	Refs.
METTL3	CRC	Anti-PD1	METTL3-single guide RNA/STM2457	MC38/CT26 allografts	Potentiates the Effect of AntiPD1 Therapy	[101]
METTL3	TNBC	Anti-PD1	STM2457	AT3 TNBC model	Improved survival for the combination of STM2457 with anti-PD1 therapy	[154]
METTL3	NAFLD-HCC	Anti-PD1	METTL3 knockdown/VNP-si METTL3/STM2457	Hepa1-6/RIL-175 tumors	Inhibiting METTL3 plus PD-1 blockade improves response to immunotherapy	[139]
METTL3		Anti-PD1	METTL3 knockout	B16 tumour	METTL3 depletion in myeloid cells impairs PD-1 blockade therapeutic efficacy	[64]
FTO		Anti-PD-L1	DAC51	B16-OVA/MC38 tumors	Slower growth of B16-OVA and MC38 tumors, and their overall survival was significantly prolonged	[132]
FTO	Melanoma	Anti-PD-1	FTO knockdown	B10F10	FTO inhibition can reduce resistance to anti-PD-1 therapy	[155]
FTO	HCC	Anti-PD-1	CS2	Orthotopic liver injection mouse model; spontaneous HCC tumours	Sensitised HCC to anti-PD-1 therapy	[142]
ALKBH5	CRC	Anti-PD1	VNP-siALKBH5	MC38 tumor	Enhances the efficacy of anti-PD1 therapy	[141]
ALKBH5	NSCLC	Anti-PD-L1	ALKBH5 knockdown	LLC allografts	Lung cancer cells with high ALKBH5 expression are more sensitive to anti-PD-L1 therapy	[103]
YTHDF1		Anti-PD1	YTHDF1 knockdown	CT26/MC38 tumours	The combination therapy prolonged OS of mice	[149]
YTHDF1		Anti-PD-L1/anti-CTLA-4	YTHDF1 knockdown	B16/F10 tumours	Tumor-intrinsic YTHDF1 deficiency enhances responses to ICI therapy	[122]
YTHDF1	CRC	Anti-PD-1	VNP-siYTHDF1	MC38/CT26 syngeneic tumours	Augments anti-PD1 therapy in CRC	[104]
YTHDF1	NASH-HCC	Anti-PD1	LNP-si YTHDF1	RIL-175 tumor	Synergistically decreased tumor burden	[99]
YTHDF2		Anti-PD-L1	DC-Y13-27	MC38/B16 tumor	The triple therapy of DC-Y13-27, IR, and anti-PD-L1 gave rise to the most robust antitumor effects	[67]
YTHDF2		Anti-PD-L1/anti-PD-1	DF-A7	MC38 tumor	Improves antitumor efficacy of PD-1/PD-L1 blockade therapy	[105]
YTHDF2	Liver tumor	Anti-PD1	YTHDF2 knockout	MC38 liver metastatic tumor	The synergistic therapeutic effects of chemotherapy and immunotherapy on liver cancer were dependent on hepatic YTHDF2 expression	[106]

VNP-siYthdf1: Vesicle-like nanoparticles (VNPs)-encapsulated YTHDF1-siRNA; CS2, a specific inhibitor of FTO; DC-Y13-27, as an inhibitor of YTHDF2; LNP-siYthdf1, lipid nanoparticles (LNP)-encapsulated siYthdf1; DF-A7, one compound mediated the degradation of YTHDF2; VNP-siALKBH5, vesicle-like nanoparticle–encapsulated ALKBH5-siRNA

In the MC38 allograft mouse model, knockout of METTL3 in tumor cells or treatment with STM2457, a highly potent and selective inhibitor of METTL3, synergistically enhances the efficacy of anti-PD-1 therapy, significantly reducing MDSC infiltration and increasing CD8 + T cell infiltration. This combination therapy demonstrates the strongest inhibitory effect on tumor growth [101]. Similar findings have been observed in the CT26 allograft mouse model [101]. In a separate study, the combination of STM2457 with anti-PD-1 therapy resulted in improved survival in mice bearing AT3 triple-negative breast cancer (TNBC) compared to single-agent therapy [154]. The combination of anti-PD-1 and STM2457 treatment also markedly increased the levels of IFN-γ + and Granzyme B + CD8 + T cells in NAFLD-HCC tumors and exhibited synergistic inhibition of tumor growth in syngeneic orthotopic NAFLD-HCC models [139]. Furthermore, depletion of Mettl3 in myeloid cells was associated with reduced responsiveness to anti-PD-1 therapy in melanoma B16 tumor metastasis models [64]. Collectively, these data support the notion that targeting METTL3 in conjunction with ICIs may provide therapeutic benefits across multiple cancer types, including CRC, TNBC, NAFLD-HCC, and melanoma.

Treatment with Dac51, a potent inhibitor of FTO, has been shown to enhance T cell infiltration and synergistically augment the effects of anti-PD-L1 blockade, as evidenced by the reduced growth of B16-OVA and MC38 tumors and the extension of overall survival [132]. Additionally, CS2, another FTO inhibitor, in combination with anti-PD-1 therapy, significantly promoted the infiltration of CD45 + F4/80 + macrophages, particularly antitumoral M1 macrophages, in spontaneous HCC tumors. Furthermore, CS2 treatment effectively suppressed tumor growth and enhanced the therapeutic efficacy of anti-PD-1 in the HCC mouse model [142].

Notably, genetic knockout of FTO in melanoma cells resulted in a marked increase in CD4 + tumor-infiltrating lymphocyte (TIL) numbers and IFN-γ production in B16F10 melanoma following anti-PD-1 blockade. Silencing of FTO further sensitized melanoma cells to IFN-γ and enhanced the response of melanoma to anti-PD-1 treatment in murine models [155]. Zhai et al. employed a virus-like nanoparticle (VNP) system to deliver specific ALKBH5 siRNAs (VNP-siALKBH5) directly into tumors. This combination achieved the most pronounced growth inhibitory effects against MC38 allografts. Consistent with these findings, treatment with VNP-siALKBH5 alongside anti-PD-1 significantly reduced intratumoral accumulation of MDSCs while increasing infiltration of CD8 + T cells and NK cells [141]. Additionally, knockdown of ALKBH5 significantly enhanced the inhibitory effect of anti-PD-L1 in LLC NSCLC tumors [103]. Collectively, these studies indicate that pharmacological inhibition or silencing of FTO and ALKBH5, two critical demethylases, can substantially sensitize cancers to ICI therapy.

Remarkably, the concurrent inhibition of m6A readers YTHDF1 and YTHDF2 in combination with ICIs may mitigate resistance to immunotherapy in cancer. Bao et al. utilized a VNP system to deliver specific Ythdf1-siRNA into tumors, successfully downregulating YTHDF1 expression within tumor tissues [104]. They further demonstrated that treatment with VNP-siYthdf1 significantly enhanced the inhibitory effects against MC38 and CT26 tumor growth following anti-PD-1 treatment. This combination therapy notably suppressed the recruitment of MDSCs and increased the tumor infiltration of IFN-γ + CD8 + T cells and Granzyme B + CD8 + T cells [104]. In a separate study, the combination of YTHDF1 knockdown and anti-PD-1 therapy significantly prolonged overall survival in mice bearing MC38 or CT26 tumors compared to those receiving either treatment alone [149]. The silencing of YTHDF1 using lipid nanoparticles encapsulated with siYthdf1 (LNP-siYthdf1), in conjunction with anti-PD-1 therapy, synergistically inhibited MDSC recruitment and activated CD8 + T cell function in mouse models of NASH-HCC, thereby reducing tumor burden and growth [99]. Furthermore, tumor-intrinsic YTHDF1 deficiency demonstrated synergistic effects with anti-CTLA-4 or anti-PD-L1 antibodies, leading to reduced tumor volume and extended overall survival in mice bearing B16/F10 cells [122]. Notably, the synergistic effects of YTHDF2 inhibition combined with ICI therapy have garnered considerable attention. DC-Y13-27, a derivative of the YTHDF2 inhibitor DC-Y13, preferentially inhibits YTHDF2 binding to m6A-modified RNA. It has been established that, compared to either monotherapy, the combination of DC-Y13-27 and anti-PD-L1 significantly slowed the growth of MC38 tumors, while the triple therapy of DC-Y13-27, radiotherapy, and anti-PD-L1 resulted in the most pronounced antitumor effects [67]. Xiao et al. validated that targeting YTHDF2 with the compound DF-A7, which mediates YTHDF2 degradation, effectively controls tumor growth and enhances antitumor efficacy when combined with anti-PD-1/PD-L1 therapy [105]. Interestingly, YTHDF2 in hepatocytes exhibited antitumor functions by modulating CX3CL1-induced CD8 + T cell infiltration. Additionally, YTHDF2 depletion in hepatocytes increased the antitumor efficacy of oxaliplatin and anti-PD-1 antibody combination therapy in the liver [106].

In summary, these findings indicate that the suppression of various m6A regulators enhances the response to ICI immunotherapy across multiple cancer types, thereby offering a promising avenue for future research in immunotherapy. It is imperative that the combination therapy targeting m6A regulators alongside ICIs be evaluated in clinical settings to assess its therapeutic efficacy.

## Conclusions and perspectives

In recent years, significant advancements have been made in the field of m6A modifications, with an increasing emphasis on the relationship between m6A modifications and tumor immunity [5, 8, 30]. This review summarizes the involvement of m6A modification in regulating immune cells within the TME through various direct and indirect mechanisms. Specifically, m6A modifications can directly influence the populations and functions of diverse immune cells, including DCs, TAMs, MDSCs, NK cells, and T cells, thereby contributing to the establishment of an immunosuppressive TME. Moreover, m6A modifications can modulate cancer cell behavior to influence the immune response by regulating cytokines, chemokines, cell surface molecules, and metabolic reprogramming. Importantly, pharmacological inhibition or silencing of various m6A regulators has been shown to enhance the response to ICI immunotherapy across different cancer types by altering the immune landscape of the TME. Collectively, these findings highlight the critical role of m6A modifications as regulators of tumor immune responses, positioning them as potential biomarkers and therapeutic targets for cancer immunotherapy.

Significantly, several challenges within this field remain to be addressed. Although numerous studies have investigated the clinical relevance of m6A regulators and the tumor immune landscape in specimens from various cancers (Table 1), these investigations have typically utilized a limited number of tumor samples, which may result in inconclusive findings. Additionally, the molecular mechanisms by which m6A-mediated posttranscriptional modifications influence immune responses in the TME have yet to be fully elucidated. For instance, beyond PD-L1, it remains unclear whether m6A modifications can regulate other immune checkpoint molecules such as B7-H3, VISTA, and B7-H4 in tumor cells. Furthermore, while researchers have focused on the synergistic effects of targeting m6A regulators in conjunction with ICI therapy, the roles of m6A modifications in other forms of cancer immunotherapy, including adoptive cell therapies and cancer vaccines, require further experimental validation. Importantly, although no clinical trials have yet evaluated m6A inhibitors for cancer treatment, combining these inhibitors with ICI therapy holds promise for enhancing anticancer efficacy. Future clinical trials are essential to substantiate these findings in human subjects.

In conclusion, our review highlights the roles and mechanisms associated with m6A modification-mediated immune responses within the TME, potentially providing a pathway for improving immunotherapy by targeting specific m6A regulators.

## Data Availability

Not applicable.

## Abbreviations

ICI: Immune checkpoint inhibitor
m6A: N6-Methyladenosine
3′UTR: 3′Untranslated region
TME: Tumor environment
METTL3: Methyltransferase-like 3
METTL14: Methyltransferase-like 14
WTAP: WT1 associated protein
RBM15/15B: RNA-binding motif protein 15/15B
VIRMA: Vir-like m6A methyltransferase associated
ZC3H13: Zinc finger CCCH-type containing 13
SAM: Sadenosylmethionine
mRNA: Messenger RNA
MAT2A: Methionine adenosyltransferase 2A
FTO: Fat mass and obesity-associated protein
ALKBH5: AlkB homolog 5
YTH: YT521-B homology
YTHDF1/2/3: YTH domain family proteins
YTHDC1/2: YTH domain-containing proteins
HNRNPC/HNRNPG/HNRNPA2B1: Heterogeneous nuclear ribonucleoproteins
IGF2BP1/2/3: Insulin-like growth factor 2 mRNA-binding proteins
eIF3: Translation initiation factor 3
RBPs: RNA-binding proteins
PRRC2A: Proline rich coiledcoil 2 A
GC: Gastric cancer
ccRCC: Clear cell renal cell carcinoma
CRC: Colorectal cancer
MDSC: Myeloid-derived suppressor cell
RIG-I: RNA sensor RIG-I
HNSCC: Head and neck squamous cell carcinoma
GBM: Glioblastoma
TAM: Tumor-associated macrophage
NSCLC: Non-small-cell lung cancer
ICC: Intrahepatic cholangiocarcinoma
HCC: Hepatocellular carcinoma
BLCA: Bladder cancer
DCs: Dendritic cells
NK cell: Natural killer cell
TIRAP: TIR domain containing adaptor protein
TLR4: Toll like receptor 4
LLC: Lewis lung carcinoma
Ebi3: Epstein-Barr virus induced 3
DUSP9: Dual specificity phosphatase 9
IRAK3: Interleukin 1 receptor associated kinase 3
SPRED2: Sprouty related EVH1 domain containing 2
BMDMs: Bone marrow-derived macrophages
BAMBI: Bone morphogenetic protein and activin membrane-bound inhibitor
K: Lysine
Jak1: Janus kinase 1
TIMs: Tumor-infiltrating myeloid cells
STAT5: Signal transducer and activator of transcription 5
Eomes: Eemesodermin
SOCS: Suppressor of cytokine signaling
USP47: Ubiquitin specific peptidase 47
THBS1: Thrombospondin 1
IFNGR1: IFN-γ receptor 1
MHC-I: Major histocompatibility complex class I
CTL: Cytotoxic T lymphocytes
DDX58: DExD/H-box helicase 58
YY1: Yin Yang 1
NASH-HCC: Non-alcoholic steatohepatitis-hepatocellular carcinoma
EZH2: Enhancer of zeste homolog 2
BHLHE41: Basic helix-loop-helix family member e41
SFPQ: Splicing factor proline and glutamine rich
VISTA: V- domain Ig suppressor of T cell activation
CBX1: Chromobox 1
NPC: Nasopharyngeal carcinoma
ETV5: ETS variant transcription factor 5
TRIM21: Tripartite motif containing 21
OTUB1: OTU deubiquitinase, ubiquitin aldehyde binding 1
PKP3: Plakophilin 3
SPOP: Speckle type BTB/POZ protein
Hsp70: Heat shock protein family A
HSPA4: Hsp70 member 4
B4LILRB4: Leukocyte immunoglobulin like receptor
PGAM1: Phosphoglycerate mutase 1
HKs: Hexokinases
RCC: Renal cell carcinoma
SREBP: Sterol regulatory element-binding protein
SCAP: SREBP cleavage-activating protein
DKK1: Dickkopf-1
GPNMB: Glycoprotein non-metastatic melanoma protein B
SDC4: Syndecan 4
TNBC: Triple-negative breast cancer
TIL: Tumor-infiltrating lymphocyte
LNP: Lipid nanoparticles

## References

[1] Gajewski TF, Schreiber H, Fu YX. Innate and adaptive immune cells in the tumor microenvironment. Nat Immunol. 2013;14:1014–22. 24048123 10.1038/ni.2703 PMC4118725

[2] Sonnenberg GF, Hepworth MR. Functional interactions between innate lymphoid cells and adaptive immunity. Nat Rev Immunol. 2019;19:599–613. 31350531 10.1038/s41577-019-0194-8 PMC6982279

[3] Vesely MD, Kershaw MH, Schreiber RD, Smyth MJ. Natural innate and adaptive immunity to cancer. Annu Rev Immunol. 2011;29:235–71. 21219185 10.1146/annurev-immunol-031210-101324

[4] Lv B, Wang Y, Ma D, Cheng W, Liu J, Yong T, et al. Immunotherapy: Reshape the Tumor Immune Microenvironment. Front Immunol. 2022;13: 844142. 35874717 10.3389/fimmu.2022.844142 PMC9299092

[5] Kong Y, Yu J, Ge S, Fan X. Novel insight into RNA modifications in tumor immunity: promising targets to prevent tumor immune escape. Innovation (Camb). 2023;4: 100452. 37485079 10.1016/j.xinn.2023.100452 PMC10362524

[6] Deng X, Qing Y, Horne D, Huang H, Chen J. The roles and implications of RNA m(6)A modification in cancer. Nat Rev Clin Oncol. 2023;20:507–26. 37221357 10.1038/s41571-023-00774-x PMC12466201

[7] Yi YC, Chen XY, Zhang J, Zhu JS. Novel insights into the interplay between m(6)A modification and noncoding RNAs in cancer. Mol Cancer. 2020;19:121. 32767982 10.1186/s12943-020-01233-2 PMC7412851

[8] Li X, Ma S, Deng Y, Yi P, Yu J. Targeting the RNA m(6)A modification for cancer immunotherapy. Mol Cancer. 2022;21:76. 35296338 10.1186/s12943-022-01558-0 PMC8924732

[9] Zhou X, Li C, Chen T, Li W, Wang X, Yang Q. Targeting RNA N6-methyladenosine to synergize with immune checkpoint therapy. Mol Cancer. 2023;22:36. 36810108 10.1186/s12943-023-01746-6 PMC9942356

[10] Zhang L, Hou C, Chen C, Guo Y, Yuan W, Yin D, et al. The role of N(6)-methyladenosine (m(6)A) modification in the regulation of circRNAs. Mol Cancer. 2020;19:105. 32522202 10.1186/s12943-020-01224-3 PMC7285594

[11] Liu Y, Yang D, Liu T, Chen J, Yu J, Yi P. N6-methyladenosine-mediated gene regulation and therapeutic implications. Trends Mol Med. 2023;29:454–67. 37068987 10.1016/j.molmed.2023.03.005

[12] Zhang X, Peng Q, Wang L. N(6)-methyladenosine modification-a key player in viral infection. Cell Mol Biol Lett. 2023;28:78. 37828480 10.1186/s11658-023-00490-5 PMC10571408

[13] Qi YN, Liu Z, Hong LL, Li P, Ling ZQ. Methyltransferase-like proteins in cancer biology and potential therapeutic targeting. J Hematol Oncol. 2023;16:89. 37533128 10.1186/s13045-023-01477-7 PMC10394802

[14] Wang X, Feng J, Xue Y, Guan Z, Zhang D, Liu Z, et al. Structural basis of N(6)-adenosine methylation by the METTL3-METTL14 complex. Nature. 2016;534:575–8. 27281194 10.1038/nature18298

[15] Yan C, Xiong J, Zhou Z, Li Q, Gao C, Zhang M, et al. A cleaved METTL3 potentiates the METTL3-WTAP interaction and breast cancer progression. Elife. 2023;12:RP87283. 37589705 10.7554/eLife.87283 PMC10435237

[16] Li N, Zhan X. Identification of pathology-specific regulators of m(6)A RNA modification to optimize lung cancer management in the context of predictive, preventive, and personalized medicine. EPMA J. 2020;11:485–504. 32849929 10.1007/s13167-020-00220-3 PMC7429590

[17] Yue Y, Liu J, Cui X, Cao J, Luo G, Zhang Z, et al. VIRMA mediates preferential m(6)A mRNA methylation in 3’UTR and near stop codon and associates with alternative polyadenylation. Cell Discov. 2018;4:10. 29507755 10.1038/s41421-018-0019-0 PMC5826926

[18] Wen J, Lv R, Ma H, Shen H, He C, Wang J, et al. Zc3h13 regulates nuclear RNA m(6)A methylation and mouse embryonic stem cell self-renewal. Mol Cell. 2018;69:1028-1038 e6. 29547716 10.1016/j.molcel.2018.02.015 PMC5858226

[19] Sun L, Zhang Y, Yang B, Sun S, Zhang P, Luo Z, et al. Lactylation of METTL16 promotes cuproptosis via m(6)A-modification on FDX1 mRNA in gastric cancer. Nat Commun. 2023;14:6523. 37863889 10.1038/s41467-023-42025-8 PMC10589265

[20] Dai Z, Zhu W, Hou Y, Zhang X, Ren X, Lei K, et al. METTL5-mediated 18S rRNA m(6)A modification promotes oncogenic mRNA translation and intrahepatic cholangiocarcinoma progression. Mol Ther. 2023;31:3225–42. 37735874 10.1016/j.ymthe.2023.09.014 PMC10638452

[21] Ren W, Lu J, Huang M, Gao L, Li D, Wang GG, et al. Structure and regulation of ZCCHC4 in m(6)A-methylation of 28S rRNA. Nat Commun. 2019;10:5042. 31695039 10.1038/s41467-019-12923-x PMC6834594

[22] Satterwhite ER, Mansfield KD. RNA methyltransferase METTL16: targets and function. Wiley Interdiscip Rev RNA. 2022;13: e1681. 34227247 10.1002/wrna.1681 PMC9286414

[23] Yoshinaga M, Han K, Morgens DW, Horii T, Kobayashi R, Tsuruyama T, et al. The N(6)-methyladenosine methyltransferase METTL16 enables erythropoiesis through safeguarding genome integrity. Nat Commun. 2022;13:6435. 36307435 10.1038/s41467-022-34078-y PMC9616860

[24] Huang J, Shao Y, Gu W. Function and clinical significance of N6-methyladenosine in digestive system tumours. Exp Hematol Oncol. 2021;10:40. 34246319 10.1186/s40164-021-00234-1 PMC8272376

[25] Bartosovic M, Molares HC, Gregorova P, Hrossova D, Kudla G, Vanacova S. N6-methyladenosine demethylase FTO targets pre-mRNAs and regulates alternative splicing and 3’-end processing. Nucleic Acids Res. 2017;45:11356–70. 28977517 10.1093/nar/gkx778 PMC5737695

[26] Zheng G, Dahl JA, Niu Y, Fedorcsak P, Huang CM, Li CJ, et al. ALKBH5 is a mammalian RNA demethylase that impacts RNA metabolism and mouse fertility. Mol Cell. 2013;49:18–29. 23177736 10.1016/j.molcel.2012.10.015 PMC3646334

[27] You XJ, Zhang S, Chen JJ, Tang F, He J, Wang J, et al. Formation and removal of 1, N6-dimethyladenosine in mammalian transfer RNA. Nucleic Acids Res. 2022;50:9858–72. 36095124 10.1093/nar/gkac770 PMC9508817

[28] Flamand MN, Tegowski M, Meyer KD. The Proteins of mRNA modification: writers, readers, and erasers. Annu Rev Biochem. 2023;92:145–73. 37068770 10.1146/annurev-biochem-052521-035330 PMC10443600

[29] Feng H, Yuan X, Wu S, Yuan Y, Cui L, Lin D, et al. Effects of writers, erasers and readers within miRNA-related m6A modification in cancers. Cell Prolif. 2023;56: e13340. 36162823 10.1111/cpr.13340 PMC9816932

[30] Li W, Hao Y, Zhang X, Xu S, Pang D. Targeting RNA N(6)-methyladenosine modification: a precise weapon in overcoming tumor immune escape. Mol Cancer. 2022;21:176. 36071523 10.1186/s12943-022-01652-3 PMC9454167

[31] Yue SW, Liu HL, Su HF, Luo C, Liang HF, Zhang BX, et al. m6A-regulated tumor glycolysis: new advances in epigenetics and metabolism. Mol Cancer. 2023;22:137. 37582735 10.1186/s12943-023-01841-8 PMC10426175

[32] Chen L, Gao Y, Xu S, Yuan J, Wang M, Li T, et al. N6-methyladenosine reader YTHDF family in biological processes: structures, roles, and mechanisms. Front Immunol. 2023;14:1162607. 36999016 10.3389/fimmu.2023.1162607 PMC10043241

[33] Sikorski V, Selberg S, Lalowski M, Karelson M, Kankuri E. The structure and function of YTHDF epitranscriptomic m(6)A readers. Trends Pharmacol Sci. 2023;44:335–53. 37069041 10.1016/j.tips.2023.03.004

[34] Zaccara S, Jaffrey SR. A unified model for the function of YTHDF proteins in regulating m(6)A-Modified mRNA. Cell. 2020;181:1582-1595 e18. 32492408 10.1016/j.cell.2020.05.012 PMC7508256

[35] Xiao W, Adhikari S, Dahal U, Chen YS, Hao YJ, Sun BF, et al. Nuclear m(6)A Reader YTHDC1 Regulates mRNA Splicing. Mol Cell. 2016;61:507–19. 26876937 10.1016/j.molcel.2016.01.012

[36] Roundtree IA, Luo GZ, Zhang Z, Wang X, Zhou T, Cui Y, et al. YTHDC1 mediates nuclear export of N(6)-methyladenosine methylated mRNAs. Elife. 2017;6: e31311. 28984244 10.7554/eLife.31311 PMC5648532

[37] Wu X, Chen H, Li K, Zhang H, Li K, Tan H. The biological function of the N6-Methyladenosine reader YTHDC2 and its role in diseases. J Transl Med. 2024;22:490. 38790013 10.1186/s12967-024-05293-6 PMC11119022

[38] Jiang F, Tang X, Tang C, Hua Z, Ke M, Wang C, et al. HNRNPA2B1 promotes multiple myeloma progression by increasing AKT3 expression via m6A-dependent stabilization of ILF3 mRNA. J Hematol Oncol. 2021;14:54. 33794982 10.1186/s13045-021-01066-6 PMC8017865

[39] Alarcon CR, Goodarzi H, Lee H, Liu X, Tavazoie S, Tavazoie SF. HNRNPA2B1 is a mediator of m(6)A-Dependent Nuclear RNA processing events. Cell. 2015;162:1299–308. 26321680 10.1016/j.cell.2015.08.011 PMC4673968

[40] Wu B, Su S, Patil DP, Liu H, Gan J, Jaffrey SR, et al. Molecular basis for the specific and multivariant recognitions of RNA substrates by human hnRNP A2/B1. Nat Commun. 2018;9:420. 29379020 10.1038/s41467-017-02770-z PMC5789076

[41] Huang H, Weng H, Sun W, Qin X, Shi H, Wu H, et al. Recognition of RNA N(6)-methyladenosine by IGF2BP proteins enhances mRNA stability and translation. Nat Cell Biol. 2018;20:285–95. 29476152 10.1038/s41556-018-0045-z PMC5826585

[42] Ramesh-Kumar D, Guil S. The IGF2BP family of RNA binding proteins links epitranscriptomics to cancer. Semin Cancer Biol. 2022;86:18–31. 35643219 10.1016/j.semcancer.2022.05.009

[43] Meyer KD, Patil DP, Zhou J, Zinoviev A, Skabkin MA, Elemento O, et al. 5’ UTR m(6)A promotes cap-independent translation. Cell. 2015;163:999–1010. 26593424 10.1016/j.cell.2015.10.012 PMC4695625

[44] Wu R, Li A, Sun B, Sun JG, Zhang J, Zhang T, et al. A novel m(6)A reader Prrc2a controls oligodendroglial specification and myelination. Cell Res. 2019;29:23–41. 30514900 10.1038/s41422-018-0113-8 PMC6318280

[45] Zhang K, Zhang Y, Maharjan Y, Sugiokto FG, Wan J, Li R. Caspases Switch off the m(6)A RNA modification pathway to foster the replication of a ubiquitous human tumor virus. MBio. 2021;12:e0170621. 34425696 10.1128/mBio.01706-21 PMC8406275

[46] Xu H, Wang H, Zhao W, Fu S, Li Y, Ni W, et al. SUMO1 modification of methyltransferase-like 3 promotes tumor progression via regulating Snail mRNA homeostasis in hepatocellular carcinoma. Theranostics. 2020;10:5671–86. 32483411 10.7150/thno.42539 PMC7254988

[47] Hou G, Zhao X, Li L, Yang Q, Liu X, Huang C, et al. SUMOylation of YTHDF2 promotes mRNA degradation and cancer progression by increasing its binding affinity with m6A-modified mRNAs. Nucleic Acids Res. 2021;49:2859–77. 33577677 10.1093/nar/gkab065 PMC7969013

[48] Sugiokto FG, Saiada F, Zhang K, Li R. SUMOylation of the m6A reader YTHDF2 by PIAS1 promotes viral RNA decay to restrict EBV replication. MBio. 2024;15:e0316823. 38236021 10.1128/mbio.03168-23 PMC10865817

[49] Hinshaw DC, Shevde LA. The tumor microenvironment innately modulates cancer progression. Cancer Res. 2019;79:4557–66. 31350295 10.1158/0008-5472.CAN-18-3962 PMC6744958

[50] Yi M, Li T, Niu M, Mei Q, Zhao B, Chu Q, et al. Exploiting innate immunity for cancer immunotherapy. Mol Cancer. 2023;22:187. 38008741 10.1186/s12943-023-01885-w PMC10680233

[51] Oliveira G, Wu CJ. Dynamics and specificities of T cells in cancer immunotherapy. Nat Rev Cancer. 2023;23:295–316. 37046001 10.1038/s41568-023-00560-y PMC10773171

[52] Ruf B, Greten TF, Korangy F. Innate lymphoid cells and innate-like T cells in cancer—at the crossroads of innate and adaptive immunity. Nat Rev Cancer. 2023;23:351–71. 37081117 10.1038/s41568-023-00562-w

[53] Damei I, Trickovic T, Mami-Chouaib F, Corgnac S. Tumor-resident memory T cells as a biomarker of the response to cancer immunotherapy. Front Immunol. 2023;14:1205984. 37545498 10.3389/fimmu.2023.1205984 PMC10399960

[54] Tran Janco JM, Lamichhane P, Karyampudi L, Knutson KL. Tumor-infiltrating dendritic cells in cancer pathogenesis. J Immunol. 2015;194:2985–91. 25795789 10.4049/jimmunol.1403134 PMC4369768

[55] Gerhard GM, Bill R, Messemaker M, Klein AM, Pittet MJ. Tumor-infiltrating dendritic cell states are conserved across solid human cancers. J Exp Med. 2021;218:1. 10.1084/jem.20200264 PMC7754678 33601412

[56] Wang H, Hu X, Huang M, Liu J, Gu Y, Ma L, et al. Mettl3-mediated mRNA m(6)A methylation promotes dendritic cell activation. Nat Commun. 2019;10:1898. 31015515 10.1038/s41467-019-09903-6 PMC6478715

[57] Han D, Liu J, Chen C, Dong L, Liu Y, Chang R, et al. Anti-tumour immunity controlled through mRNA m(6)A methylation and YTHDF1 in dendritic cells. Nature. 2019;566:270–4. 30728504 10.1038/s41586-019-0916-x PMC6522227

[58] Li M, Yang Y, Xiong L, Jiang P, Wang J, Li C. Metabolism, metabolites, and macrophages in cancer. J Hematol Oncol. 2023;16:80. 37491279 10.1186/s13045-023-01478-6 PMC10367370

[59] Cassetta L, Pollard JW. A timeline of tumour-associated macrophage biology. Nat Rev Cancer. 2023;23:238–57. 36792751 10.1038/s41568-022-00547-1

[60] Shu Y, Cheng P. Targeting tumor-associated macrophages for cancer immunotherapy. Biochim Biophys Acta Rev Cancer. 2020;1874: 188434. 32956767 10.1016/j.bbcan.2020.188434

[61] Dong L, Chen C, Zhang Y, Guo P, Wang Z, Li J, et al. The loss of RNA N(6)-adenosine methyltransferase Mettl14 in tumor-associated macrophages promotes CD8(+) T cell dysfunction and tumor growth. Cancer Cell. 2021;39:945-957 e10. 34019807 10.1016/j.ccell.2021.04.016

[62] Guo X, Qiu W, Li B, Qi Y, Wang S, Zhao R, et al. Hypoxia-induced neuronal activity in glioma patients polarizes microglia by potentiating RNA m6A demethylation. Clin Cancer Res. 2024;30:1160–74. 37855702 10.1158/1078-0432.CCR-23-0430

[63] Tong J, Wang X, Liu Y, Ren X, Wang A, Chen Z, et al. Pooled CRISPR screening identifies m(6)A as a positive regulator of macrophage activation. Sci Adv. 2021;7:eabd4742. 33910903 10.1126/sciadv.abd4742 PMC8081357

[64] Yin H, Zhang X, Yang P, Zhang X, Peng Y, Li D, et al. RNA m6A methylation orchestrates cancer growth and metastasis via macrophage reprogramming. Nat Commun. 2021;12:1394. 33654093 10.1038/s41467-021-21514-8 PMC7925544

[65] Lasser SA, Ozbay Kurt FG, Arkhypov I, Utikal J, Umansky V. Myeloid-derived suppressor cells in cancer and cancer therapy. Nat Rev Clin Oncol. 2024;21:147–64. 38191922 10.1038/s41571-023-00846-y

[66] De Sanctis F, Solito S, Ugel S, Molon B, Bronte V, Marigo I. MDSCs in cancer: conceiving new prognostic and therapeutic targets. Biochim Biophys Acta. 2016;1865:35–48. 26255541 10.1016/j.bbcan.2015.08.001

[67] Wang L, Dou X, Chen S, Yu X, Huang X, Zhang L, et al. YTHDF2 inhibition potentiates radiotherapy antitumor efficacy. Cancer Cell. 2023;41:1294-1308 e8. 37236197 10.1016/j.ccell.2023.04.019 PMC10524856

[68] Wang L, Si W, Yu X, Piffko A, Dou X, Ding X, et al. Epitranscriptional regulation of TGF-beta pseudoreceptor BAMBI by m6A/YTHDF2 drives extrinsic radioresistance. J Clin Invest. 2023;133: e172919. 38099498 10.1172/JCI172919 PMC10721150

[69] Zhang D, Tang Z, Huang H, Zhou G, Cui C, Weng Y, et al. Metabolic regulation of gene expression by histone lactylation. Nature. 2019;574:575–80. 31645732 10.1038/s41586-019-1678-1 PMC6818755

[70] Xiong J, He J, Zhu J, Pan J, Liao W, Ye H, et al. Lactylation-driven METTL3-mediated RNA m(6)A modification promotes immunosuppression of tumor-infiltrating myeloid cells. Mol Cell. 2022;82:1660-1677 e10. 35320754 10.1016/j.molcel.2022.02.033

[71] Shimasaki N, Jain A, Campana D. NK cells for cancer immunotherapy. Nat Rev Drug Discov. 2020;19:200–18. 31907401 10.1038/s41573-019-0052-1

[72] Cantoni C, Falco M, Vitale M, Pietra G, Munari E, Pende D, et al. Human NK cells and cancer. Oncoimmunology. 2024;13:2378520. 39022338 10.1080/2162402X.2024.2378520 PMC11253890

[73] Ma S, Yan J, Barr T, Zhang J, Chen Z, Wang LS, et al. The RNA m6A reader YTHDF2 controls NK cell antitumor and antiviral immunity. J Exp Med. 2021;218: e20210279. 34160549 10.1084/jem.20210279 PMC8225680

[74] Song H, Song J, Cheng M, Zheng M, Wang T, Tian S, et al. METTL3-mediated m(6)A RNA methylation promotes the anti-tumour immunity of natural killer cells. Nat Commun. 2021;12:5522. 34535671 10.1038/s41467-021-25803-0 PMC8448775

[75] Dong C. Cytokine regulation and function in T Cells. Annu Rev Immunol. 2021;39:51–76. 33428453 10.1146/annurev-immunol-061020-053702

[76] O’Donnell JS, Teng MWL, Smyth MJ. Cancer immunoediting and resistance to T cell-based immunotherapy. Nat Rev Clin Oncol. 2019;16:151–67. 30523282 10.1038/s41571-018-0142-8

[77] Choi Y, Shi Y, Haymaker CL, Naing A, Ciliberto G, Hajjar J. T-cell agonists in cancer immunotherapy. J Immunother Cancer. 2020;8:1. 10.1136/jitc-2020-000966 PMC7537335 33020242

[78] Li HB, Tong J, Zhu S, Batista PJ, Duffy EE, Zhao J, et al. m(6)A mRNA methylation controls T cell homeostasis by targeting the IL-7/STAT5/SOCS pathways. Nature. 2017;548:338–42. 28792938 10.1038/nature23450 PMC5729908

[79] Tong J, Cao G, Zhang T, Sefik E, Amezcua Vesely MC, Broughton JP, et al. m(6)A mRNA methylation sustains Treg suppressive functions. Cell Res. 2018;28:253–6. 29303144 10.1038/cr.2018.7 PMC5799823

[80] Wang A, Huang H, Shi JH, Yu X, Ding R, Zhang Y, et al. USP47 inhibits m6A-dependent c-Myc translation to maintain regulatory T cell metabolic and functional homeostasis. J Clin Invest. 2023;133:1. 10.1172/JCI169365 PMC10688989 37788092

[81] Silva-Santos B, Mensurado S, Coffelt S. B gammadelta T cells: pleiotropic immune effectors with therapeutic potential in cancer. Nat Rev Cancer. 2019;19:392–404. 31209264 10.1038/s41568-019-0153-5 PMC7614706

[82] Li J, Feng H, Zhu J, Yang K, Zhang G, Gu Y, et al. Gastric cancer derived exosomal THBS1 enhanced Vgamma9Vdelta2 T-cell function through activating RIG-I-like receptor signaling pathway in a N6-methyladenosine methylation dependent manner. Cancer Lett. 2023;576: 216410. 37783390 10.1016/j.canlet.2023.216410

[83] Bryushkova EA, Mushenkova NV, Turchaninova MA, Lukyanov DK, Chudakov DM, Serebrovskaya EO. B cell clonality in cancer. Semin Immunol. 2024;72: 101874. 38508089 10.1016/j.smim.2024.101874

[84] Burger JA, Wiestner A. Targeting B cell receptor signalling in cancer: preclinical and clinical advances. Nat Rev Cancer. 2018;18:148–67. 29348577 10.1038/nrc.2017.121

[85] Laumont CM, Banville AC, Gilardi M, Hollern DP, Nelson BH. Tumour-infiltrating B cells: immunological mechanisms, clinical impact and therapeutic opportunities. Nat Rev Cancer. 2022;22:414–30. 35393541 10.1038/s41568-022-00466-1 PMC9678336

[86] Li X, Zheng M, Ma S, Nie F, Yin Z, Liang Y, et al. YTHDC1 is a therapeutic target for B-cell acute lymphoblastic leukemia by attenuating DNA damage response through the KMT2C-H3K4me1/me3 epigenetic axis. Leukemia. 2024;2024:1–15. 10.1038/s41375-024-02451-z 39501105

[87] Meng S, Xia Y, Li M, Wu Y, Wang D, Zhou Y, et al. NCBP1 enhanced proliferation of DLBCL cells via METTL3-mediated m6A modification of c-Myc. Sci Rep. 2023;13:8606. 37244946 10.1038/s41598-023-35777-2 PMC10224985

[88] Zhou B, Chen N, Chen Z, Chen S, Yang J, Zheng Y, et al. Prmt5 deficient mouse B cells display RNA processing complexity and slower colorectal tumor progression. Eur J Immunol. 2023;53: e2250226. 37389889 10.1002/eji.202250226

[89] Zhao L, Guo J, Xu S, Duan M, Liu B, Zhao H, et al. Abnormal changes in metabolites caused by m(6)A methylation modification: the leading factors that induce the formation of immunosuppressive tumor microenvironment and their promising potential for clinical application. J Adv Res. 2024;2024:1.10.1016/j.jare.2024.04.01638677545

[90] Lukhele S, Rabbo DA, Guo M, Shen J, Elsaesser HJ, Quevedo R, et al. The transcription factor IRF2 drives interferon-mediated CD8(+) T cell exhaustion to restrict anti-tumor immunity. Immunity. 2022;55:2369-2385 e10. 36370712 10.1016/j.immuni.2022.10.020 PMC9809269

[91] Di Franco S, Turdo A, Todaro M, Stassi G. Role of type I and II interferons in colorectal cancer and melanoma. Front Immunol. 2017;8:878. 28798748 10.3389/fimmu.2017.00878 PMC5526853

[92] Jorgovanovic D, Song M, Wang L, Zhang Y. Roles of IFN-gamma in tumor progression and regression: a review. Biomark Res. 2020;8:49. 33005420 10.1186/s40364-020-00228-x PMC7526126

[93] You Q, Wang F, Du R, Pi J, Wang H, Huo Y, et al. m(6) A Reader YTHDF1-targeting engineered small extracellular vesicles for gastric cancer therapy via epigenetic and immune regulation. Adv Mater. 2023;35: e2204910. 36484103 10.1002/adma.202204910

[94] Zhang Y, Liu Z, Zhong Z, Ji Y, Guo H, Wang W, et al. A tumor suppressor protein encoded by circKEAP1 inhibits osteosarcoma cell stemness and metastasis by promoting vimentin proteasome degradation and activating anti-tumor immunity. J Exp Clin Cancer Res. 2024;43:52. 38383479 10.1186/s13046-024-02971-7 PMC10880370

[95] Borden EC. Interferons alpha and beta in cancer: therapeutic opportunities from new insights. Nat Rev Drug Discov. 2019;18:219–34. 30679806 10.1038/s41573-018-0011-2

[96] Jin S, Li M, Chang H, Wang R, Zhang Z, Zhang J, et al. The m6A demethylase ALKBH5 promotes tumor progression by inhibiting RIG-I expression and interferon alpha production through the IKKepsilon/TBK1/IRF3 pathway in head and neck squamous cell carcinoma. Mol Cancer. 2022;21:97. 35395767 10.1186/s12943-022-01572-2 PMC8994291

[97] Zhang L, Li Y, Zhou L, Zhou H, Ye L, Ou T, et al. The m6A Reader YTHDF2 promotes bladder cancer progression by suppressing RIG-I-Mediated immune response. Cancer Res. 2023;83:1834–50. 36939388 10.1158/0008-5472.CAN-22-2485 PMC10236158

[98] Qiu Z, Zhao L, Shen JZ, Liang Z, Wu Q, Yang K, et al. Transcription elongation machinery is a druggable dependency and potentiates immunotherapy in glioblastoma stem cells. Cancer Discov. 2022;12:502–21. 34615656 10.1158/2159-8290.CD-20-1848 PMC8831451

[99] Wang L, Zhu L, Liang C, Huang X, Liu Z, Huo J, et al. Targeting N6-methyladenosine reader YTHDF1 with siRNA boosts antitumor immunity in NASH-HCC by inhibiting EZH2-IL-6 axis. J Hepatol. 2023;79:1185–200. 37459919 10.1016/j.jhep.2023.06.021

[100] Ozga AJ, Chow MT, Luster AD. Chemokines and the immune response to cancer. Immunity. 2021;54:859–74. 33838745 10.1016/j.immuni.2021.01.012 PMC8434759

[101] Chen H, Pan Y, Zhou Q, Liang C, Wong CC, Zhou Y, et al. METTL3 inhibits antitumor immunity by targeting m(6)A-BHLHE41-CXCL1/CXCR2 axis to promote colorectal cancer. Gastroenterology. 2022;163:891–907. 35700773 10.1053/j.gastro.2022.06.024

[102] Dong F, Qin X, Wang B, Li Q, Hu J, Cheng X, et al. ALKBH5 facilitates hypoxia-induced paraspeckle assembly and IL8 secretion to generate an immunosuppressive tumor microenvironment. Cancer Res. 2021;81:5876–88. 34670781 10.1158/0008-5472.CAN-21-1456

[103] Hua X, Xu Q, Wu R, Sun W, Gu Y, Zhu S, et al. ALKBH5 promotes non-small cell lung cancer progression and susceptibility to anti-PD-L1 therapy by modulating interactions between tumor and macrophages. J Exp Clin Cancer Res. 2024;43:164. 38872221 10.1186/s13046-024-03073-0 PMC11177518

[104] Bao Y, Zhai J, Chen H, Wong CC, Liang C, Ding Y, et al. Targeting m(6)A reader YTHDF1 augments antitumour immunity and boosts anti-PD-1 efficacy in colorectal cancer. Gut. 2023;72:1497–509. 36717220 10.1136/gutjnl-2022-328845 PMC10359538

[105] Xiao S, Ma S, Sun B, Pu W, Duan S, Han J, et al. The tumor-intrinsic role of the m(6)A reader YTHDF2 in regulating immune evasion. Sci Immunol. 2024;9: eadl2171. 38820140 10.1126/sciimmunol.adl2171 PMC12068375

[106] Yang Z, Wang X, Fu Y, Wu W, Hu Z, Lin Q, et al. YTHDF2 in peritumoral hepatocytes mediates chemotherapy-induced antitumor immune responses through CX3CL1-mediated CD8(+) T cell recruitment. Mol Cancer. 2024;23:186. 39237909 10.1186/s12943-024-02097-6 PMC11378438

[107] Burke KP, Chaudhri A, Freeman GJ, Sharpe AH. The B7:CD28 family and friends: unraveling coinhibitory interactions. Immunity. 2024;57:223–44. 38354702 10.1016/j.immuni.2024.01.013 PMC10889489

[108] Borgeaud M, Sandoval J, Obeid M, Banna G, Michielin O, Addeo A, et al. Novel targets for immune-checkpoint inhibition in cancer. Cancer Treat Rev. 2023;120: 102614. 37603905 10.1016/j.ctrv.2023.102614

[109] Cha JH, Chan LC, Li CW, Hsu JL, Hung MC. Mechanisms controlling PD-L1 expression in cancer. Mol Cell. 2019;76:359–70. 31668929 10.1016/j.molcel.2019.09.030 PMC6981282

[110] Luo P, Li S, Long X. N6-methyladenosine RNA modification in PD-1/PD-L1: novel implications for immunotherapy. Biochim Biophys Acta Rev Cancer. 2023;1878: 188873. 36842764 10.1016/j.bbcan.2023.188873

[111] Liu L, Liang L, Li H, Shao W, Yang C, Lin F, et al. The role of m6A-mediated PD-1/PD-L1 in antitumor immunity. Biochem Pharmacol. 2023;210: 115460. 36822438 10.1016/j.bcp.2023.115460

[112] Wan W, Ao X, Chen Q, Yu Y, Ao L, Xing W, et al. METTL3/IGF2BP3 axis inhibits tumor immune surveillance by upregulating N(6)-methyladenosine modification of PD-L1 mRNA in breast cancer. Mol Cancer. 2022;21:60. 35197058 10.1186/s12943-021-01447-y PMC8864846

[113] Ni Z, Sun P, Zheng J, Wu M, Yang C, Cheng M, et al. JNK signaling promotes bladder cancer immune escape by regulating METTL3-Mediated m6A modification of PD-L1 mRNA. Cancer Res. 2022;82:1789–802. 35502544 10.1158/0008-5472.CAN-21-1323

[114] Qiu X, Yang S, Wang S, Wu J, Zheng B, Wang K, et al. M(6)A demethylase ALKBH5 regulates PD-L1 expression and tumor immunoenvironment in intrahepatic cholangiocarcinoma. Cancer Res. 2021;81:4778–93. 34301762 10.1158/0008-5472.CAN-21-0468

[115] Zhao Y, Huang S, Tan X, Long L, He Q, Liang X, et al. N(6) -Methyladenosine-Modified CBX1 regulates nasopharyngeal carcinoma progression through heterochromatin formation and STAT1 activation. Adv Sci (Weinh). 2022;9: e2205091. 36310139 10.1002/advs.202205091 PMC9798977

[116] Wen J, Xue L, Wei Y, Liang J, Jia W, Yong T, et al. YTHDF2 is a therapeutic target for HCC by suppressing immune evasion and angiogenesis through ETV5/PD-L1/VEGFA axis. Adv Sci (Weinh). 2024;11: e2307242. 38247171 10.1002/advs.202307242 PMC10987122

[117] Liu Y, Guo Q, Yang H, Zhang XW, Feng N, Wang JK, et al. Allosteric regulation of IGF2BP1 as a novel strategy for the activation of tumor immune microenvironment. ACS Cent Sci. 2022;8:1102–15. 36032766 10.1021/acscentsci.2c00107 PMC9413439

[118] Sun Z, Mai H, Xue C, Fan Z, Li J, Chen H, et al. Hsa-LINC02418/mmu-4930573I07Rik regulated by METTL3 dictates anti-PD-L1 immunotherapeutic efficacy via enhancement of Trim21-mediated PD-L1 ubiquitination. J Immunother Cancer. 2023;11:1. 38040417 10.1136/jitc-2023-007415 PMC10693898

[119] Liu Z, Wang T, She Y, Wu K, Gu S, Li L, et al. N(6)-methyladenosine-modified circIGF2BP3 inhibits CD8(+) T-cell responses to facilitate tumor immune evasion by promoting the deubiquitination of PD-L1 in non-small cell lung cancer. Mol Cancer. 2021;20:105. 34416901 10.1186/s12943-021-01398-4 PMC8377850

[120] Li J, Xu X, Xu K, Zhou X, Wu K, Yao Y, et al. N6-methyladenosine-modified circSLCO1B3 promotes intrahepatic cholangiocarcinoma progression via regulating HOXC8 and PD-L1. J Exp Clin Cancer Res. 2024;43:119. 38641828 10.1186/s13046-024-03006-x PMC11031933

[121] Sari G, Rock KL. Tumor immune evasion through loss of MHC class-I antigen presentation. Curr Opin Immunol. 2023;83: 102329. 37130455 10.1016/j.coi.2023.102329 PMC10524158

[122] Lin W, Chen L, Zhang H, Qiu X, Huang Q, Wan F, et al. Tumor-intrinsic YTHDF1 drives immune evasion and resistance to immune checkpoint inhibitors via promoting MHC-I degradation. Nat Commun. 2023;14:265. 36650153 10.1038/s41467-022-35710-7 PMC9845301

[123] Arulanandam AR, Withka JM, Wyss DF, Wagner G, Kister A, Pallai P, et al. The CD58 (LFA-3) binding site is a localized and highly charged surface area on the AGFCC’C” face of the human CD2 adhesion domain. Proc Natl Acad Sci USA. 1993;90:11613–7. 7505442 10.1073/pnas.90.24.11613 PMC48034

[124] Suo D, Gao X, Chen Q, Zeng T, Zhan J, Li G, et al. HSPA4 upregulation induces immune evasion via ALKBH5/CD58 axis in gastric cancer. J Exp Clin Cancer Res. 2024;43:106. 38589927 10.1186/s13046-024-03029-4 PMC11000359

[125] Deng M, Gui X, Kim J, Xie L, Chen W, Li Z, et al. LILRB4 signalling in leukaemia cells mediates T cell suppression and tumour infiltration. Nature. 2018;562:605–9. 30333625 10.1038/s41586-018-0615-z PMC6296374

[126] Sharma N, Atolagbe OT, Ge Z, Allison JP. LILRB4 suppresses immunity in solid tumors and is a potential target for immunotherapy. J Exp Med. 2021;218: e20201811. 33974041 10.1084/jem.20201811 PMC8117208

[127] Su R, Dong L, Li Y, Gao M, Han L, Wunderlich M, et al. Targeting FTO suppresses cancer stem cell maintenance and immune evasion. Cancer Cell. 2020;38(79–96): e11. 10.1016/j.ccell.2020.04.017 PMC7363590 32531268

[128] An Y, Duan H. The role of m6A RNA methylation in cancer metabolism. Mol Cancer. 2022;21:14. 35022030 10.1186/s12943-022-01500-4 PMC8753874

[129] Xia L, Oyang L, Lin J, Tan S, Han Y, Wu N, et al. The cancer metabolic reprogramming and immune response. Mol Cancer. 2021;20:28. 33546704 10.1186/s12943-021-01316-8 PMC7863491

[130] Paul S, Ghosh S, Kumar S. Tumor glycolysis, an essential sweet tooth of tumor cells. Semin Cancer Biol. 2022;86:1216–30. 36330953 10.1016/j.semcancer.2022.09.007

[131] Ganapathy-Kanniappan S. Linking tumor glycolysis and immune evasion in cancer: Emerging concepts and therapeutic opportunities. Biochim Biophys Acta Rev Cancer. 2017;1868:212–20. 28400131 10.1016/j.bbcan.2017.04.002

[132] Liu Y, Liang G, Xu H, Dong W, Dong Z, Qiu Z, et al. Tumors exploit FTO-mediated regulation of glycolytic metabolism to evade immune surveillance. Cell Metab. 2021;33:1221-1233 e11. 33910046 10.1016/j.cmet.2021.04.001

[133] Huang K, Liang Q, Zhou Y, Jiang LL, Gu WM, Luo MY, et al. A novel allosteric inhibitor of phosphoglycerate mutase 1 suppresses growth and metastasis of non-small-cell lung cancer. Cell Metab. 2019;30(1107–1119): e8. 10.1016/j.cmet.2019.09.014 31607564

[134] Liu Z, Zheng N, Li J, Li C, Zheng D, Jiang X, et al. N6-methyladenosine-modified circular RNA QSOX1 promotes colorectal cancer resistance to anti-CTLA-4 therapy through induction of intratumoral regulatory T cells. Drug Resist Updat. 2022;65: 100886. 36370665 10.1016/j.drup.2022.100886

[135] Seiler K, Humbert M, Minder P, Mashimo I, Schlafli AM, Krauer D, et al. Hexokinase 3 enhances myeloid cell survival via non-glycolytic functions. Cell Death Dis. 2022;13:448. 35538058 10.1038/s41419-022-04891-w PMC9091226

[136] Zhang Y, Chen M, Liu M, Xu Y, Wu G. Glycolysis-related genes serve as potential prognostic biomarkers in clear cell renal cell carcinoma. Oxid Med Cell Longev. 2021;2021:6699808. 33564363 10.1155/2021/6699808 PMC7850857

[137] Li T, Gu Y, Xu B, Kuca K, Zhang J, Wu W. CircZBTB44 promotes renal carcinoma progression by stabilizing HK3 mRNA structure. Mol Cancer. 2023;22:77. 37106446 10.1186/s12943-023-01771-5 PMC10134651

[138] King RJ, Singh PK, Mehla K. The cholesterol pathway: impact on immunity and cancer. Trends Immunol. 2022;43:78–92. 34942082 10.1016/j.it.2021.11.007 PMC8812650

[139] Pan Y, Chen H, Zhang X, Liu W, Ding Y, Huang D, et al. METTL3 drives NAFLD-related hepatocellular carcinoma and is a therapeutic target for boosting immunotherapy. Cell Rep Med. 2023;4: 101144. 37586322 10.1016/j.xcrm.2023.101144 PMC10439254

[140] Shi T, Zhang Y, Wang Y, Song X, Wang H, Zhou X, et al. DKK1 promotes tumor immune evasion and impedes Anti-PD-1 treatment by inducing immunosuppressive macrophages in gastric cancer. Cancer Immunol Res. 2022;10:1506–24. 36206576 10.1158/2326-6066.CIR-22-0218

[141] Zhai J, Chen H, Wong CC, Peng Y, Gou H, Zhang J, et al. ALKBH5 drives immune suppression via targeting AXIN2 to promote colorectal cancer and is a target for boosting immunotherapy. Gastroenterology. 2023;165:445–62. 37169182 10.1053/j.gastro.2023.04.032

[142] Chen A, Zhang VX, Zhang Q, Sze KM, Tian L, Huang H, et al. Targeting the oncogenic m6A demethylase FTO suppresses tumourigenesis and potentiates immune response in hepatocellular carcinoma. Gut. 2024;74:90–102. 38839271 10.1136/gutjnl-2024-331903 PMC11672076

[143] Pan J, Huang T, Deng Z, Zou C. Roles and therapeutic implications of m6A modification in cancer immunotherapy. Front Immunol. 2023;14:1132601. 36960074 10.3389/fimmu.2023.1132601 PMC10028070

[144] Gan L, Zhao Y, Fu Y, Chen Q. The potential role of m6A modifications on immune cells and immunotherapy. Biomed Pharmacother. 2023;160: 114343. 36758318 10.1016/j.biopha.2023.114343

[145] Chong W, Shang L, Liu J, Fang Z, Du F, Wu H, et al. m(6)A regulator-based methylation modification patterns characterized by distinct tumor microenvironment immune profiles in colon cancer. Theranostics. 2021;11:2201–17. 33500720 10.7150/thno.52717 PMC7797678

[146] Zhang B, Wu Q, Li B, Wang D, Wang L, Zhou YL. m(6)A regulator-mediated methylation modification patterns and tumor microenvironment infiltration characterization in gastric cancer. Mol Cancer. 2020;19:53. 32164750 10.1186/s12943-020-01170-0 PMC7066851

[147] Zhong J, Liu Z, Cai C, Duan X, Deng T, Zeng G. m(6)A modification patterns and tumor immune landscape in clear cell renal carcinoma. J Immunother Cancer. 2021;9:1. 10.1136/jitc-2020-001646 PMC7880120 33574053

[148] Bai X, Wong CC, Pan Y, Chen H, Liu W, Zhai J, et al. Loss of YTHDF1 in gastric tumors restores sensitivity to antitumor immunity by recruiting mature dendritic cells. J Immunother Cancer. 2022;10:1. 10.1136/jitc-2021-003663 PMC9066370 35193930

[149] Li T, Tan YT, Chen YX, Zheng XJ, Wang W, Liao K, et al. Methionine deficiency facilitates antitumour immunity by altering m(6)A methylation of immune checkpoint transcripts. Gut. 2023;72:501–11. 35803704 10.1136/gutjnl-2022-326928 PMC9933173

[150] Kennedy LB, Salama AKS. A review of cancer immunotherapy toxicity. CA Cancer J Clin. 2020;70:86–104. 31944278 10.3322/caac.21596

[151] Riley RS, June CH, Langer R, Mitchell MJ. Delivery technologies for cancer immunotherapy. Nat Rev Drug Discov. 2019;18:175–96. 30622344 10.1038/s41573-018-0006-z PMC6410566

[152] Darvin P, Toor SM, Sasidharan Nair V, Elkord E. Immune checkpoint inhibitors: recent progress and potential biomarkers. Exp Mol Med. 2018;50:1–11. 30546008 10.1038/s12276-018-0191-1 PMC6292890

[153] Igarashi Y, Sasada T. Cancer vaccines: toward the next breakthrough in cancer immunotherapy. J Immunol Res. 2020;2020:5825401. 33282961 10.1155/2020/5825401 PMC7685825

[154] Guirguis AA, Ofir-Rosenfeld Y, Knezevic K, Blackaby W, Hardick D, Chan YC, et al. Inhibition of METTL3 results in a cell-intrinsic interferon response that enhances antitumor immunity. Cancer Discov. 2023;13:2228–47. 37548590 10.1158/2159-8290.CD-23-0007

[155] Yang S, Wei J, Cui YH, Park G, Shah P, Deng Y, et al. m(6)A mRNA demethylase FTO regulates melanoma tumorigenicity and response to anti-PD-1 blockade. Nat Commun. 2019;10:2782. 31239444 10.1038/s41467-019-10669-0 PMC6592937

